# Molecular mechanism of the wake-promoting agent TAK-925

**DOI:** 10.1038/s41467-022-30601-3

**Published:** 2022-05-25

**Authors:** Jie Yin, Yanyong Kang, Aaron P. McGrath, Karen Chapman, Megan Sjodt, Eiji Kimura, Atsutoshi Okabe, Tatsuki Koike, Yuhei Miyanohana, Yuji Shimizu, Rameshu Rallabandi, Peng Lian, Xiaochen Bai, Mack Flinspach, Jef K. De Brabander, Daniel M. Rosenbaum

**Affiliations:** 1 Department of Biophysics, The University of Texas Southwestern Medical Center, 6001 Forest Park Road, Dallas, TX 75390 USA; 2 Takeda Development Center Americas, Inc, 9625 Towne Centre Drive, San Diego, CA 92121 USA; 3 Takeda Pharmaceutical Company Ltd., 26-1 Muraoka-Higashi, 2-Chome, Fujisawa, Kanagawa 251-8555 Japan; 4 Department of Biochemistry, The University of Texas Southwestern Medical Center, Dallas, TX 75390 USA; 5 BioHPC at the Lyda Hill Department of Bioinformatics, The University of Texas Southwestern Medical Center, Dallas, TX 75390 USA; 6 Present Address: Chinese Institute for Brain Research, No. 26 Science Park Road, Zhongguancun Life Science Park, Changping District, Beijing, China

**Keywords:** Cryoelectron microscopy, Receptor pharmacology, G protein-coupled receptors

## Abstract

The OX_2_ orexin receptor (OX_2_R) is a highly expressed G protein-coupled receptor (GPCR) in the brain that regulates wakefulness and circadian rhythms in humans. Antagonism of OX_2_R is a proven therapeutic strategy for insomnia drugs, and agonism of OX_2_R is a potentially powerful approach for narcolepsy type 1, which is characterized by the death of orexinergic neurons. Until recently, agonism of OX_2_R had been considered ‘undruggable.’ We harness cryo-electron microscopy of OX_2_R-G protein complexes to determine how the first clinically tested OX_2_R agonist TAK-925 can activate OX_2_R in a highly selective manner. Two structures of TAK-925-bound OX_2_R with either a G_q_ mimetic or G_i_ reveal that TAK-925 binds at the same site occupied by antagonists, yet interacts with the transmembrane helices to trigger activating microswitches. Our structural and mutagenesis data show that TAK-925’s selectivity is mediated by subtle differences between OX_1_ and OX_2_ receptor subtypes at the orthosteric pocket. Finally, differences in the polarity of interactions at the G protein binding interfaces help to rationalize OX_2_R’s coupling selectivity for G_q_ signaling. The mechanisms of TAK-925’s binding, activation, and selectivity presented herein will aid in understanding the efficacy of small molecule OX_2_R agonists for narcolepsy and other circadian disorders.

## Abstract (web summary)

The OX_2_ orexin receptor (OX_2_R) is a brain GPCR that regulates wakefulness and circadian rhythms in humans, and a potential drug target in insomnia and narcolepsy. Here, the authors use cryo-EM to determine how the first clinically tested OX_2_R agonist TAK-925 can activate OX_2_R in a selective manner.

## Introduction

Orexin signaling in the brain is the primary mechanism connecting diurnal circadian rhythms to arousal and wakefulness. Orexin A and B are neuropeptides (33 and 28 amino acids respectively, also known as hypocretins) derived from the preproorexin precursor and produced by a small set of dedicated neurons in the lateral hypothalamus, which stimulate release of neurotransmitters in diverse brain regions to promote wakefulness^1^. Orexin release is controlled by the circadian clock, and levels of orexin neuropeptides in mammalian cerebrospinal fluid follow a 24-hour cycle, peaking during the wake period^2,3^. The orexin peptides act by binding to the GPCRs OX1R and OX2R on target neurons to activate cellular signaling by G proteins and arrestins, particularly stimulating Gq/11-mediated release of calcium from the endoplasmic reticulum^4^.

The human disorder narcolepsy type 1 is characterized by a deficiency of orexin signaling, resulting in a pentad of symptoms including excessive daytime sleepiness, disturbed nighttime sleep, hypnagogic/hypnopompic hallucinations, sleep paralysis, and cataplexy, a sudden loss of muscle tone triggered by strong emotions^5^. Narcolepsy type 1 affects approximately 100,000 adults in the U.S^6^., and up to 3 million patients may be affected by the disorder worldwide. While the etiology of this disorder varies, the proximal cause of narcolepsy type 1 is the death of orexin neurons^7^. The resulting loss of orexin expression in the narcoleptic brain has been validated in human clinical studies^8^, and the causal relationship between orexin deficiency and the narcolepsy phenotype has been validated using preproorexin knockout mice and targeted killing of the orexin neurons^9,10^.

Experiments in canine and mouse models also established that orexin control of circadian rhythms and wakefulness occurs predominantly through the actions of OX_2_R^11,12^, while signaling through OX_1_R is critical for stimulation of reward pathways^13^. These discoveries paved the way for small molecule drug discovery efforts targeting the orexin receptors in sleep/wake disorders, culminating in FDA approval of the dual orexin receptor antagonist (DORA) suvorexant (Belsomra®) for insomnia in 2014^14^. Numerous small molecule chemotypes have been developed as DORAs or OX_2_R-selective antagonists^15^, however the discovery of small molecule OX_2_R agonists has lagged far behind, with very few potent lead compounds reported in the primary literature^16^ or patents. A breakthrough in this area was recently achieved with the small molecule TAK-925^17^, which displays low-nanomolar potency for OX_2_R activation and >5000-fold selectivity for OX_2_R over OX_1_R^18^. In animal studies, subcutaneous delivery of TAK-925 during the sleep period results in a dose-dependent increase in wakefulness and reduction in sleep time. TAK-925 is currently being explored as an orexin agonist in humans with narcolepsy, with multiple trials ongoing.

Insights into the molecular basis for orexin receptor activation and inhibition have come from structural studies of OX_1_R and OX_2_R bound to different ligands. The first structure of OX_2_R with suvorexant revealed that the drug binds in a narrow membrane-embedded pocket analogous to the classic orthosteric site of adrenaline and beta-blocker binding in the β_2_-adrenergic receptor^19^. Structures of OX_1_R and OX_2_R with other antagonists showed that these compounds invariably bind to the same suvorexant site^20–22^. However, mutagenesis studies of the orexin receptors and other neuropeptide-activated GPCRs showed that interactions of the peptide agonists with the solvent-exposed extracellular loops (notably ECL2) are required to trigger full activation^23^, possibly underlying the difficulty in identifying small molecule agonist drug candidates. A recent cryo-EM study showed how a small molecule could mimic orexin B (OxB) to stabilize an active conformation of OX_2_R bound to a G protein^24^.

In this work, we use cryo-EM of different OX_2_R-G protein complexes and associated pharmacological studies to understand the molecular mechanism of the drug candidate TAK-925. These structural and functional data reveal how TAK-925 activates OX_2_R with high potency and subtype selectivity, and with a preference for signaling through G_q_.

## Results

### Structure determination of TAK-925-bound OX_2_R coupled to G_q_ and G_i1_

**Fig. 1:**
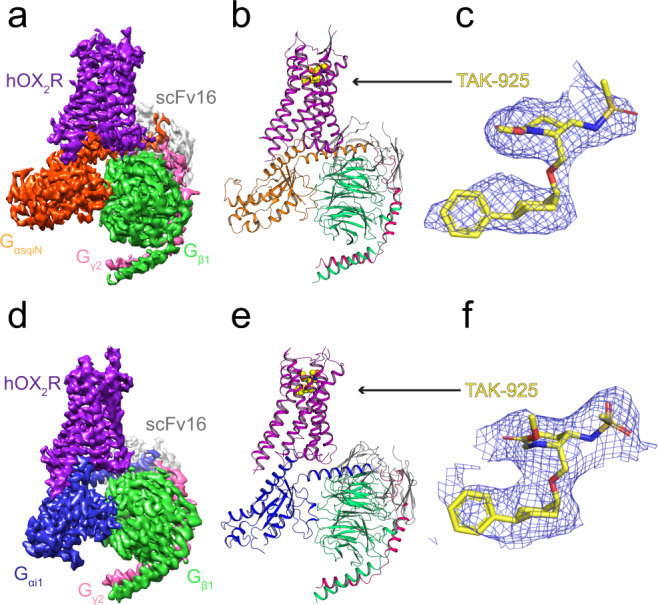
Overall structures of the OX_2_R-G protein complexes. **a** Cryo-EM reconstruction of the complex OX_2_R-mG_sqiN_. Receptor density is in purple, TAK-925 is yellow, G_αsqiN_ is orange, G_β1_ is green, G_γ2_ is pink. The cryo-EM density from Relion was displayed in Chimera as colored surfaces (contour level 0.033, 5 sigma), where different colored zones corresponded to the different polypeptides. **b** Model of complex as a cartoon, with the TAK-925 agonist as spheres with yellow carbons, blue nitrogens and red oxygens. **c** Cryo-EM density surrounding the ligand TAK-925 in the complex structure OX_2_R-mG_sqiN_, with the TAK-925 agonist as sticks with colored by heteroatom (yellow carbons, blue nitrogens and red oxygens). Cryo-EM density was displayed in Pymol (4.2 sigma) within 2 Å around TAK-925. **d** Cryo-EM reconstruction of the complex OX_2_R-G_i1_. Receptor density is in purple, TAK-925 is yellow, G_αi1_ is blue, G_β1_ is green, G_γ2_ is pink. The cryo-EM density from cryoSPARC was displayed in Chimera as colored surfaces (contour level 0.5, 5 sigma), where different colored zones corresponded to the different polypeptides. **e** Model of complex as a cartoon, with the TAK-925 agonist as spheres with yellow carbons, blue nitrogens and red oxygens. **f** Cryo-EM density surrounding the ligand TAK-925 in the complex structure OX_2_R-G_i1_, with the TAK-925 agonist as sticks with colored by heteroatom (yellow carbons, blue nitrogens and red oxygens). Cryo-EM density was displayed in Pymol (5 sigma) within 2 Å around TAK-925.

To capture active structures of OX2R bound to TAK-925, we purified complexes of the receptor together with heterotrimeric G proteins. Using recombinant protein from Sf9 insect cells, we elucidated the cryo-EM structure of OX_2_R coupled to a mini-G_αs/q/iN_/G_β_/G_γ_ heterotrimer (referred to as OX_2_R-mG_sqiN_ in this manuscript) in lauryl maltose neopentyl glycol (LMNG) micelles at 3.3 Å resolution (Supplementary Figs. 1a, b, and 2), serving as a model for the G_q_ signaling complex leading to calcium release. In parallel, we solved the cryo-EM structure of OX_2_R coupled to a DNG_αi1_/G_β_/G_γ_ heterotrimer (referred to as OX_2_R-G_i1_) in digitonin at 3.2 Å resolution (Supplementary Figs. 1c, d, and 3), representing a G_i_ signaling complex. Both cryo-EM efforts took advantage of scFv16 binding^25^, requiring generation of a modified mini-G_s/q_71^26^ construct capable of binding the antibody fragment. The global cryo-EM envelope, atomic model, and density for TAK-925 in OX_2_R-mG_sqiN_ are shown in Fig. 1a–c, while the analogous data for OX_2_R-G_i1_ are in Fig. 1d–f. Differences between OX_2_R models in these two structures are greatest at the G protein interface, although the models of the agonist-bound receptor are generally in close agreement, with a root mean square deviation (rmsd) of 0.7 Å. Both cryo-EM maps have well-defined sidechain density in the transmembrane region (Supplementary Fig. 4), and allowed placement and refinement of TAK-925 (Fig. 1c, f). The stability of the modeled ligand conformation, particular regarding the saturated rings, was validated by quantum chemistry calculations (Supplementary Fig. 5).

### Binding of TAK-925 to OX_2_R

**Fig. 2:**
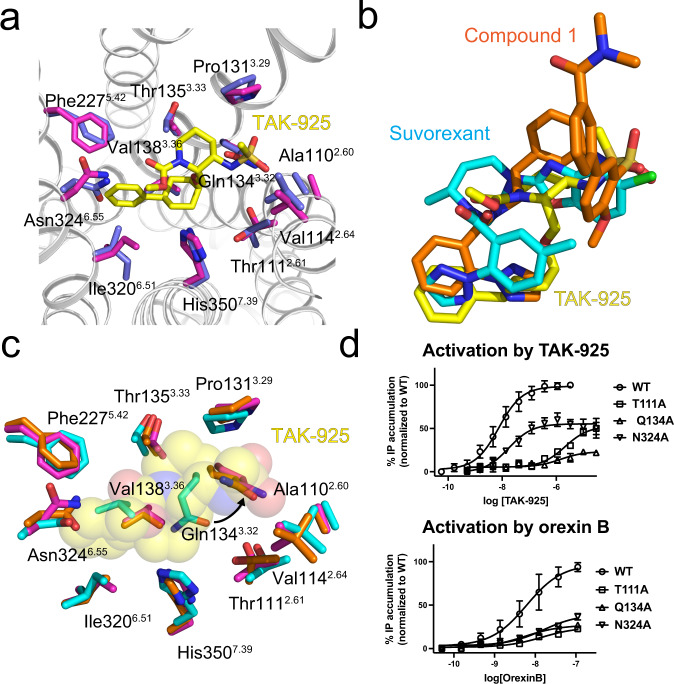
Binding of TAK-925 to OX_2_R. **a** Overlay of contact residues (sticks with purple carbons for mG_sqiN_-coupled OX_2_R and sticks with blue carbons for G_i1_-coupled OX_2_R) within 4 Å of TAK-925 (yellow carbons) when superimposing OX_2_R- mG_sqiN_ and OX_2_R-G_i1_. The OX_2_R backbone (silver) is from OX_2_R-mG_sqiN_. The hydrogen bond from Gln134^3.32^ to TAK-925’s sulfonamide is not shown because this residue is behind the ligand from this viewpoint (same as in Fig. 2c). **b** Overlay of TAK-925 (sticks with yellow carbons), Compound 1 (sticks with orange carbons) and suvorexant (sticks with cyan carbons) when superimposing the OX_2_R polypeptides from the OX_2_R-mG_sqiN_ complex (this work), the OX_2_R-mini-G_sqi_ complex (PDB 7L1V) and the antagonist-bound inactive conformation (PDB 4S0V). **c** Overlay of contact residues within 4 Å of TAK-925 when superimposing OX_2_R-mG_sqiN_ (this work, magenta), OX_2_R-mini-G_sqi_/Compound 1 (PDB 7L1V, orange) and the suvorexant-bound inactive conformation of OX_2_R (PDB 4S0V, cyan). TAK-925 is shown as transparent spheres. **d** Stimulation of G_q_ by OX_2_R wild type (WT) and mutants when bound to TAK-925 (top) and orexin B (bottom). Each data point represents an average from *n* ≥ 3 independent experiments (each performed in duplicate), where n is shown in Supplementary Table 2. Error bars are ±SD. Data were normalized to the WT E_max_ and fitted to the three-parameter model ‘log(agonist) vs response’ in GraphPad Prism 9. Source data are provided as a Source Data file.

The molecular interactions between TAK-925 and OX_2_R are highly similar in the mG_sqiN_ and G_i1_ complexes (Fig. 2a), which solidifies the interpretation of the details of these interfaces. The cryo-EM density for the ligand binding pockets in the OX_2_R-mG_sqiN_ and OX_2_R-G_i1_ complexes are shown in Supplementary Fig. 6a and b, respectively. TAK-925 adopts a compact, U-shaped conformation, contacting OX_2_R residues on TM2, TM3, TM5, TM6 and TM7 and burying 460 Å^2^ of solvent-accessible surface area. The methyl carbamate and sulfonamide ‘arms’ of TAK-925 extend toward polar residues on either side of the orthosteric pocket, the latter group engaging in a hydrogen bond with Gln134^3.32^, while the phenyl-cyclohexane ‘tail’ projects deeper into the transmembrane region contacting Val138^3.36^, Phe227^5.42^, and Ile320^6.51^. TAK-925 and suvorexant share an overlapping binding site in OX_2_R despite having opposing ligand efficacy (Fig. 2b). The diarylsulfonamide agonist Compound 1, reported in a previous cryo-EM structure^24^, shares elements of the TAK-925 pocket, but also extends further toward the extracellular loops of the receptor. Modulation of the position of Gln134^3.32^ was previously observed in the OX_2_R-Compound 1 and OX_2_R-OxB complexes, and we similarly find that Gln134^3.32^ undergoes the largest conformational switch of the orthosteric pocket residues with TAK-925 bound, compared to the suvorexant-bound inactive state (Fig. 2c). Despite their very different chemotypes, TAK-925 and Compound 1 both have sulfonamides at similar positions in the active OX_2_R complexes (Fig. 2b), which interact with Gln134^3.32^ and promote a shifted position of TM3 (Fig. 2c). The overall structure of active OX_2_R with TAK-925 is similar to active OX_2_R with Compound 1, with an rmsd of 0.6 Å, despite the use of a stabilizing extracellular nanobody in the Compound 1 complex.

**Fig. 3:**
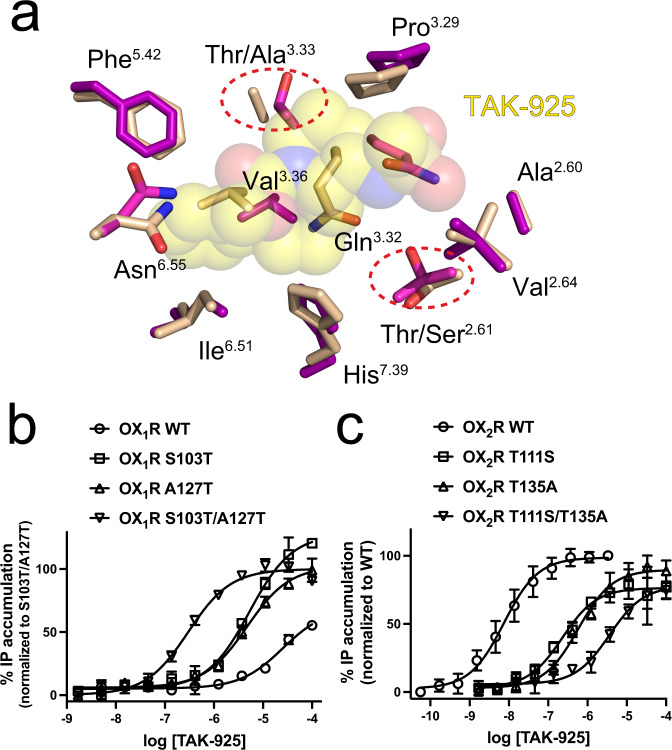
Structural basis for high selectivity of TAK-925 for OX_2_R. **a** Overlay of contact residues (sticks with purple carbons for mG_sqiN_-coupled OX_2_R and sticks with wheat carbons for inactive OX_1_R) within 4 Å of TAK-925 (transparent spheres with yellow carbons) when superimposing OX_2_R-G_q_ and the antagonist-bound inactive conformation of OX_1_R (PDB 4ZJ8). Divergent residues are circled by red dotted lines. **b** Stimulation of G_q_ by OX_1_R wild type (WT) and subtype-swap single and double mutants when bound to TAK-925. Each data point represents an average from *n* = 3 independent experiments (each performed in duplicate). Error bars are ±SD. OX_1_R data were normalized to S103T/A127T E_max_. Data were fitted to the three-parameter model ‘log(agonist) vs response’ in GraphPad Prism 9. Source data are provided as a Source Data file. **c** Stimulation of G_q_ by OX_2_R wild type (WT) and subtype-swap single and double mutants. Each data point represents an average from *n* ≥ 3 independent experiments (each performed in duplicate), where *n* is shown in Supplementary Table 2. Error bars are ±SD. OX_2_R data were normalized to OX_2_R WT E_max_. Data were fitted to the three-parameter model ‘log(agonist) vs response’ in GraphPad Prism 9. Source data are provided as a Source Data file.

To further establish that the conformational switch seen for Gln134^3.32^ is important for receptor activation, we carried out mutagenesis and measured G_q_ signaling in an inositol phosphate (IP) accumulation assay (Fig. 2d, Supplementary Table 2). Consistent with the present and previous^24^ structures, OX_2_R Q134^3.32^A loses almost all responsiveness to TAK-925 and OxB. In contrast, the N324^6.55^A mutant receptor can be activated by TAK-925, with an EC_50_ (18 nM) close to the wild-type receptor (7.5 nM) in this assay (Fig. 2d, top panel), although this mutant has a lower E_max_ likely due to lower plasma membrane expression (Supplementary Fig. 7). In the structure of OX_2_R-G_i1_, N324^6.55^ hydrogen-bonds to TAK-925’s carbonyl group with a distance of 2.7 Å between heteroatoms, however in OX_2_R-mG_sqiN_ this distance is 3.3 Å. The non-ideal hydrogen bond between the ligand and OX_2_R helps explain why this contact is largely dispensable for TAK-925 signaling through G_q_ (Fig. 3a). Unlike TAK-925, OxB fails to activate the N324^6.55^A mutant (Fig. 2d, bottom panel), and the OX_2_R-OxB-G_q_ complex^24^ previously showed that the neuropeptide residue Thr27 makes a specific hydrogen bond to Asn324^6.55^. This polar residue was also previously demonstrated to be essential for antagonist binding and inhibition of OX_2_R in structural^19,20^ and mutagenesis^27^ studies. The residue Thr111^2.61^ interacts with the ligand in the OX_2_R-mG_sqiN_ structure such that the sidechain oxygen is 3.8 Å from TAK-925’s sulfonamide nitrogen. While the structure does not indicate a hydrogen bond between these groups, we found that mutation of this residue to alanine led to significantly reduced potency of the agonist (Fig. 2d, top panel, EC_50_ 2.1 μM). This loss of activity could be due to solvent-mediated effects, as discussed below. Finally, we carried out β-arrestin recruitment assays for a panel of OX_2_R mutants, and found that the residues important for TAK-925 activation of this pathway agree with the interactions that TAK-925 makes with the active OX_2_R orthosteric pocket in the cryo-EM structures (Supplementary Fig. 8, Supplementary Tables 3 and 4). Notably, large reductions in β-arrestin recruitment by TAK-925 were measured for residues Gln134^3.32^ as well as Tyr317^6.48^ at the base of the orthosteric pocket, both of which undergo changes from the inactive to active conformations.

From ligand structure-activity relationship (SAR) studies^17^, we found that critical determinants of TAK-925’s potency include the stereochemistry of substituents on the cyclohexane and piperidine rings, which enforces TAK-925’s stable U-shaped conformation seen in our structures (Fig. 1c, f, Supplementary Fig. 5). Furthermore, the sulfonamide of the ligand is essential for potency, and this group forms a tight polar interaction with the important residue Gln134^3.32^ in the active state (Fig. 2a, c). The SAR around the phenyl ring is narrow and replacement with aliphatic groups results in a reduction in potency, which is consistent with the phenyl-cyclohexyl tail of TAK-925 projecting into the deep hydrophobic pocket lined by nonpolar residues from TMs 3, 5, and 6.

### Selectivity of TAK-925 for OX_2_R over OX_1_R

**Fig. 4:**
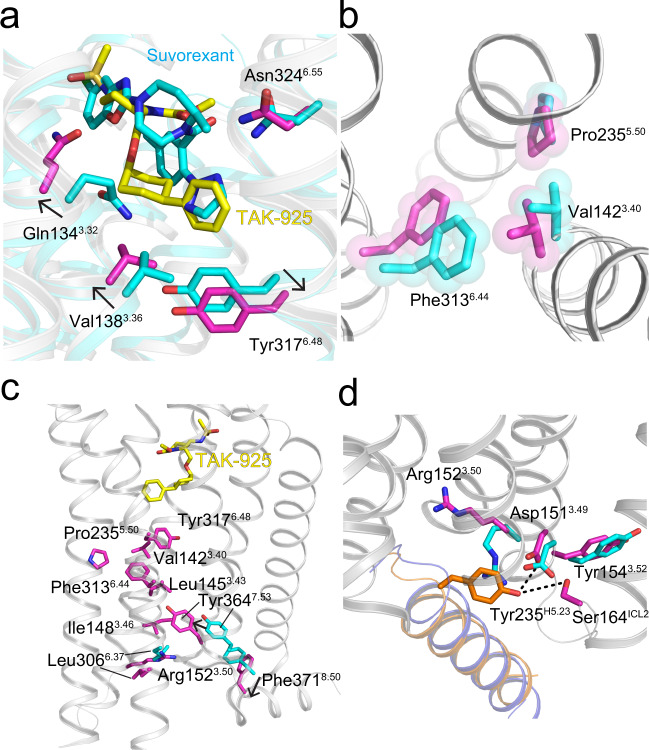
Propagated changes in OX_2_R activation. **a** Conformational changes of the side chains of key residues at the orthosteric site when bound to agonist, comparing the active conformation (purple sticks, transparent gray cartoon) and the inactive conformation (cyan sticks and transparent cyan cartoon). TAK-925 is shown as yellow sticks and suvorexant is in cyan sticks. **b** Overlay of PIF transmission switch in the active conformation of OX_2_R (purple sticks and transparent spheres) and the inactive conformation of OX_2_R (PDB 4S0V, cyan sticks and transparent spheres). **c** Rewiring of micro switches on the intracellular side of OX_2_R when bound to agonist, comparing the active conformation (purple sticks, gray cartoon) and the inactive conformation (cyan sticks). TAK-925 is shown as yellow sticks. **d** Conformational changes of DRY motif when coupled to G protein, comparing the active conformation (gray cartoon and purple stick) and the inactive conformation (cyan sticks). The H5 helices of G_q_ and G_i_ are shown as orange and blue cartoon, respectively. Hydrogen bonds are shown as dotted lines.

We have previously shown that TAK-925 has >5000 fold selectivity for OX_2_R over OX_1_R in calcium mobilization assays^18^. Our complex structures with TAK-925 show that, as with the DORA suvorexant^19,20^, the agonist contact sphere is highly conserved in OX_1_R, with only two substitutions: Thr111^2.61^→Ser and Thr135^3.33^→Ala (Fig. 3a). It is possible that selectivity is enforced locally by these small differences in the respective binding pockets, however selectivity could also be the result of longer-range allosteric differences. To distinguish these possibilities, we carried out subtype swap experiments similar to what we previously performed for OX_1_R- and OX_2_R-selective antagonists^20^. When we mutated the two divergent binding pocket residues in OX_1_R to the corresponding amino acid in OX_2_R and carried out IP accumulation assays, we observed saturable activation by TAK-925 (EC_50_ = 300 nM versus >100 μM non-saturating for wild-type OX_1_R). The two single mutants showed a partial increase (left-ward shift) in potency, indicating that both positions contribute towards selectivity (Fig. 3b). Conversely, mutation of these two positions in OX_2_R to the corresponding amino acids in OX_1_R leads to a reduction (right-ward shift) in potency, and the double mutant results in EC_50_ 3.6 μM versus 7.5 nM for wild-type OX_2_R (Fig. 4c). We also found that when OX_2_R Thr111^2.61^ is mutated to alanine (instead of serine), receptor activation by TAK-925 is diminished (Fig. 2d top panel, EC_50_ 2.1 μM). Collectively, these data indicate that the high selectivity of TAK-925 for OX_2_R is largely caused by differences in the deep orthosteric pockets of the two receptor subtypes, rather than in the more divergent extracellular loops. If the active OX_1_R adopted a different conformation at the deep orthosteric pocket where TAK-925 binds, it would not be possible to rescue signaling with the S103T/A127T double mutant. Likewise, this result indicates that the allosteric pathway of activation for OX_1_R can be turned on by agonist binding at this site, and rules out the possibility that TAK-925 has minimal potency at OX_1_R due to differences in the allosteric transmission mechanism.

### TAK-925 activation of OX_2_R

How does TAK-925 stabilize the active conformation of OX_2_R? Compared to the inactive state, binding of TAK-925 causes a rotation in TM3 at the orthosteric pocket with sidechains moving towards TM2, including the shift of Gln134^3.32^ described above. The ‘pull’ on TM3 occurs together with an opposing downward ‘push’ on Tyr317^6.48^ induced by the deeper projection (relative to suvorexant) of TAK-925’s phenyl-cyclohexyl tail against TMs 3 and 6 (Fig. 4a). This conformational change is disfavored by suvorexant due to the position of its benzoxazole, which packs against TM3 and prevents its rotation. A downward shift in the aromatic residue at position 6.48 (Ballesteros–Weinstein numbering^28^) is observed in several active GPCRs, and has been suggested to initialize the hallmark outward rotation of TM6 during GPCR activation^29^. The signal is then further propagated down the receptor, where the ‘PIF switch’ (PVF in OX_2_R)^30^ adopts an active conformation (Fig. 4b), and the vacancy left by TM6’s outward movement is filled by the inward movement of TM7 toward TM3. In particular, the microswitch residue Tyr364^7.53^ moves ~4 Å inward (Fig. 4c) and sits between residues Leu145^3.43^, Ile148^3.46^, and Arg152^3.50^, while another microswitch residue Leu306^6.37^ moves ~5 Å outward as a result of the repositioning of Arg152^3.50^. In parallel with the TM3-TM6-TM7 repacking, the ionic interaction between Asp151^3.49^ and Arg152^3.50^ is broken, and the cytosolic side of the receptor opens to accommodate the G protein α5 helix (Fig. 4d). These latter conformational changes are also seen in other GPCRs^31^.

### OX_2_R-G protein interaction

**Fig. 5:**
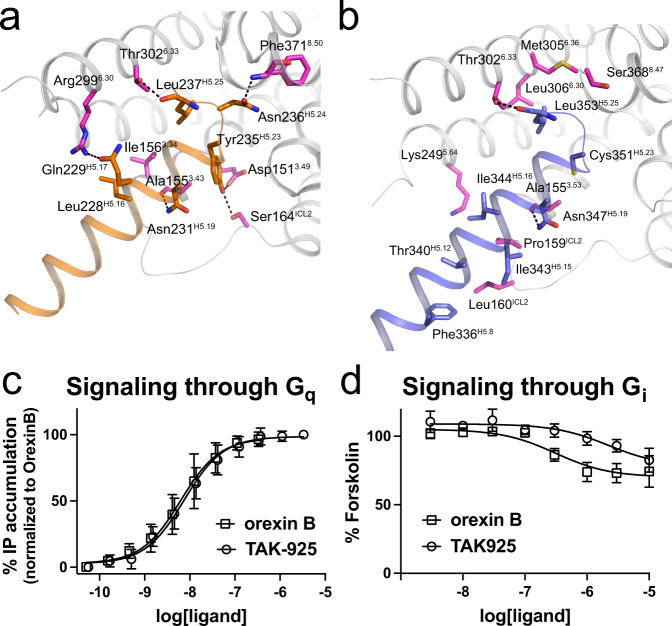
Interfaces of OX_2_R-G protein complexes and activation of G_i_ and G_q_. **a** Interactions within 4 Å between OX_2_R and mG_sqiN_ (gray cartoon and purple sticks for OX_2_R and orange cartoon and sticks for H5 helix of G_q_ α subunit). Hydrogen bonds are dotted lines. **b** Interactions within 4 Å between OX_2_R and G_i1_ (gray cartoon and purple sticks for OX_2_R and blue cartoon and sticks for H5 helix of G_i_ α subunit). Hydrogen bonds are dotted lines. **c** G_q_ signaling mediated by OX_2_R with TAK-925 or orexin B. Each data point represents an average from *n* ≥ 3 independent experiments (each performed in duplicate), where *n* is shown in Supplementary Table 2. Error bars are ±SD. Data were normalized to the orexin B E_max_ and fitted to the three-parameter model ‘log(agonist) vs response’ in GraphPad Prism 9. Source data are provided as a Source Data file. **d** G_i_ signaling mediated by OX_2_R with TAK-925 or orexin B. Each data point represents an average from 3 independent experiments (each performed in duplicate). Error bars are ±SD. Source data are provided as a Source Data file.

A major signaling cascade activated by the orexin neuropeptides in neurons is G_q_-mediated calcium release^32^. We were able to characterize TAK-925-bound samples of active OX_2_R with both mG_sqiN_ (a G_q_ mimetic) and G_i1_, and are thus able to compare the interfaces between these complexes. The cryo-EM density for the receptor-G protein interface in the OX_2_R-mG_sqiN_ and OX_2_R-G_i1_ complexes are shown in Supplementary Fig. 6c and d, respectively. A majority of the contacts occur between OX_2_R and the C-terminal α5 helix of each G protein (Fig. 5a, b), as seen in previous activated receptor complexes^25,33^. The OX_2_R-mG_sqiN_ interface has features similar to previously characterized GPCR-G_q/11_ complexes^34–37^. In particular, the end of the C-terminal α5 helix forms a ‘hook’ that packs against the TM7-Helix8 junction, and the sidechain of Tyr235^H5.23^ from this hook extends across the center of the interface to form a hydrogen bond with ICL2 of the receptor (Ser164^ICL2^ in OX_2_R, Figs. 4d and 5a). A similar interaction has been observed in other GPCR-G_q_ complexes^36,37^, and Tyr235^H5.23^ is not conserved in G_i_.

The overall conformation of the OX_2_R-G_i1_ interface is similar to that of OX_2_R-mG_sqiN_ (rmsd for receptor and Gα subunits together equals 1.3 Å). However, the OX_2_R-mG_sqiN_ interface features more extensive contacts between OX_2_R and the mG_αsqiN_, resulting in burial of more surface area (1039 Å^2^ versus 814 Å^2^), Notably, the interaction of OX_2_R with the G_i1_ α5 helix is predominantly mediated by hydrophobic contacts, and G_i1_ makes only two polar contacts to the receptor’s polypeptide backbone (Fig. 5b). In contrast, active OX_2_R makes 7 specific polar interactions with the mG_αsqiN_ α5 helix, including from Ser164^ICL2^ to Tyr253^H5.23^ and from Thr302^6.33^ to the mG_αsqiN_ C-terminus (Fig. 5a, Supplementary Figs. 9 and 10). The backbone amide of Phe371^8.50^ also hydrogen-bonds to Asn244^H5.24^ of mG_αsqiN_ (common G_α_ numbering system^38^) (Fig. 5a), and this OX_2_R residue undergoes a downward shift during activation concomitant with the upward movement of Tyr364^7.53^ (Fig. 4c).

To confirm whether OX_2_R can signal effectively through both G protein complexes, we carried out G_q_ and G_i_ signaling assays in HEK293 cells transfected with OX_2_R and G proteins. Both orexin B and TAK-925 showed poor ability to stimulate G_i1_ as measured by reduction in cAMP levels after forskolin treatment (Fig. 5d). In contrast, both orexin B and TAK-925 induced robust G_q_-mediated IP accumulation with EC_50_ values consistent with previous studies (8.2 nM and 8.1 nM, respectively) (Fig. 5c). These results show that TAK-925 is similar to OxB in selectively activating G_q_ signaling in cells.

## Discussion

TAK-925 can fully activate OX_2_R despite its low molecular weight and non-peptidic structure. Earlier mutagenesis studies showed that residues on the ECL2 β-hairpin of OX_2_R are important for activation by OxB^23^, and we previously found that an α-helix preceding TM1 is involved in orexin binding^20^, which helps to position the neuropeptide C-terminal region to interact with the membrane-embedded site including N324^6.55^. These findings made it difficult to imagine how a drug-like small molecule could recapitulate the interactions needed to activate OX_2_R. Our present structures of active OX_2_R with TAK-925, along with the recent structure with Compound 1^24^, explain how a low-MW drug can activate the receptor (Fig. 5d) without fully mimicking the orexin neuropeptide. Indeed, TAK-925 binds in the same deep membrane-embedded site as the inhibitor suvorexant (Fig. 2a, b), but is able to engage in concerted interactions with the receptor that stabilize the active conformation (Figs. 2c and 4a). The deep burial of TAK-925 at the membrane-embedded site also facilitates high affinity and potency, which the orexin neuropeptides can only achieve through multivalent contacts analogous to Class B GPCR/peptide interactions^39^. The mechanism of TAK-925 activation of OX_2_R sets a precedent that small molecule drug-like full agonists are possible even for the most challenging peptide-activated GPCR targets.

Subtype selectivity remains a major challenge in GPCR drug development, both for agonists and antagonists^40,41^. TAK-925 has a high degree of OX_2_R selectivity, and this profile may be important to avoid OX_1_R activation of brain reward pathways associated with addiction^13^, while maintaining desirable sleep/wake effects. A typical strategy for achieving GPCR ligand selectivity is to exploit subpockets of the orthosteric site that are not conserved between subtypes, or to focus on more highly divergent allosteric sites^42^. Our structures of TAK-925-bound active OX_2_R and functional data (Fig. 3) show that subtype selectivity can be achieved even when the binding pockets are extremely highly conserved (19 out of 21 residues within 5 Å of TAK-925 are identical, with only Thr→Ser and Thr→Ala substitutions). How then does TAK-925 bind so much weaker to OX_1_R? As with OX_2_R-selective antagonists such as EMPA^21^, the contact sphere surrounding the ligand does not have to be very different between subtypes. Instead, we propose that small cavities resulting from the two smaller amino acids in a potential OX_1_R/TAK-925 complex lead to subtly diminished steric complementarity and less favorable desolvation upon surface burial^43^, with a large effect on binding free energy. These cavities are filled in the OX_1_R S103^2.61^T/A127^3.33^T double swap mutant, allowing for activation of this receptor by TAK-925.

Other selective small molecule OX_2_R agonists such as Compound 1^24^ and the structurally similar YNT-185^16^ may also rely on the subtle differences between orthosteric pockets that are important for TAK-925, however the structure of Compound 1-bound OX_2_R shows that this type of diarylsulfonamide also makes contacts with the more divergent extracellular loops. Intriguingly, a recent medicinal chemistry effort^44^ succeeded in making a potent dual orexin receptor agonist by modifying YNT-185 on the biphenyl moiety that is predicted to interact with the extracellular loops (given the structural analogy with Compound 1). This finding implies that OX_2_R-selective arylsulfonamide agonists may derive selectivity from interactions that are far from the TAK-925 interface. Determining the importance of different contacts for selectivity of this other agonist chemotype will require additional functional studies of mutant receptors. In the case of the antagonist EMPA, a previous structural and computational study^22^ found that binding of a trapped ordered water molecule in a cavity formed at A127^3.33^ of the putative OX_1_R complex is a major determinant of OX_2_R selectivity for this ligand. A similar phenomenon may occur to make TAK-925’s interaction with OX_1_R unfavorable, and will require computational approaches to elucidate. Our recent demonstration^45^ of converting a dual selective antagonist (suvorexant) into an OX_1_R-selective antagonist by filling in this cavity with an aliphatic group suggests that modifying TAK-925 may be a viable strategy for creating OX_1_R-selective agonists.

Few GPCRs have been rigorously characterized for their ability to activate multiple G protein classes, such as β_2_-adrenergic receptor coupling to G_s_ and G_i_^46^. Several studies of OX_2_R using cAMP sensors or other engineered reporter assays have indicated that OX_2_R can also stimulate G_s_ and G_i_ signaling, although with reduced orexin potency^47,48^. From these studies, one may ask whether OX_2_R has any preference for activating a particular signaling cascade in cells or in vivo. Our structural data indicates that OX_2_R distinguishes between G_q_ and G_i_, both in the extent of interaction and in the number of specific polar contacts (Fig. 5a, b, Supplementary Figs. 9 and 10). Meanwhile, our functional data shows that OX_2_R cannot substantially activate G_i_ in HEK293 cells (Fig. 5d), however the caveat remains that we did not measure this activity in transfected neurons. These results bolster observations that orexins function mainly by stimulating calcium release through activating G_q_^32,49^, and suggest that G protein promiscuity plays a limited role in orexin signaling.

Several examples have recently been described of activated GPCR structures in complex with multiple G proteins (or G protein mimetics), which can provide insights into G protein selectivity. In most cases, the G protein known to couple most efficiently with the receptor has a larger buried surface area at the interface relative to subordinate G protein transducers^37,50^, which holds true for OX_2_R in this study (see above). The multivalent interactions between the receptor and the G protein are divergent, and selectivity is largely dependent on the unique features of each GPCR. In the GCGR-G_s_ complex, ICL2 of the receptor engages more closely with G_s_ compared to G_i_, and this interaction was demonstrated to be important for preferred G_s_ coupling^50^. On the other hand, the GCGR-G_i_ complex features unique contacts between ICL1 and G_β_ and between ICL3 and G_iα_^50^. In the CCK1R-G_q_ complex, ICL3 interacts with G_αq_ and was shown to be important for G_q_ coupling potency^37^. We do not observe a similar ICL3-G protein interaction in either of our OX_2_R-G protein complexes (Fig. 5a, b), and the tip of ICL3 is disordered in both structures, however the caveat remains that we have used a G_q_ mimetic (mG_sqiN_) rather than wild-type G_q_ protein in our study. In another example of multiple CCK1R-G protein structures, a different conformation was observed for the G protein α5 helix between mG_sqi_ and G_s_ complexes, which may reflect the potential for structural differences or dynamics of this key divergent G protein epitope to confer selectivity^36^. In our two OX_2_R-G protein structures, we observe highly similar conformations of the α5 helix (including the C-terminal ‘hook’) for the mG_sqi_ and G_i1_ complexes (Fig. 5a, b, rmsd of 1.3 Å as described above). On the other hand, we find that our OX_2_R-G_i1_ interface is dominated by hydrophobic contacts, and the OX_2_R-G_sqiN_ interface is a mixture of polar and hydrophobic interactions (Fig. 5a, b, Supplementary Figs. 9 and 10). A similar differentiation of interface properties is seen in comparing the preferential GCGR-G_s_ complex to the GCGR-G_i_ complex^50^. Intriguingly, several of the hydrogen bonds between OX_2_R and the mG_sqiN_ α5 helix are conserved in CCK1R-G_q_ structures:^36,37^ Y235^H5.23^-S164^ICL2^ and N231^H5.19^-A155^3.53^ from OX_2_R-mG_sqiN_ are analogous to Y391^H5.23^-Q153^ICL2^ and N387^H5.19^-A142^3.53^ in CCK1R-G_q_. This similarity may represent a shared contributor to G_q_ selectivity for multiple GPCRs.

An optimal drug for narcolepsy type 1 patients would be an oral pill that substitutes for deficient orexin neuropeptides and restores normal circadian patterns of orexin signaling, reversing debilitating symptoms such as sleep attacks and cataplexy^5,8^. This goal presents a complex set of pharmacokinetics challenges, since the drug must be orally bioavailable, brain penetrant, and have clearance kinetics on the order of hours. TAK-925 is administered intravenously (ClinicalTrials.gov NCT04091438), and more recently other small molecules have been discovered with improved oral absorption (ClinicalTrials.gov NCT04096560). The structures of active OX_2_R bound to TAK-925 presented here will help in understanding the important interactions that must be maintained to potently and fully activate the receptor by small molecule agonists.

## Methods

### Chemicals

The IUPAC name for TAK-925 is: [methyl (2*R*,3*S*)−3-[(methylsulfonyl)amino]−2-{[(*cis*−4-phenylcyclohexyl)oxy]methyl}piperidine-1-carboxylate]. TAK-925 was synthesized by Takeda Pharmaceutical Company Limited or according to U.S. patent US20170226137. Compound was characterized as follows:

mp: 113 °C; [α]D = + 16.3 (c 0.1, chloroform); 1H NMR (600 MHz, DMSO-d6): δ 1.40–1.55 (5H, m), 1.56–1.73 (5H, m), 1.87 (1H, brd, *J* = 13.2 Hz), 1.96 (1H, brd, *J* = 13.6 Hz), 2.44–2.57 (1H, m), 2.83 (1H, brs), 2.95 (3H, s), 3.40 (1H, brs), 3.53–3.62 (5H, m), 3.73 (1H, brt, *J* = 9.7 Hz), 3.84 (1H, brs), 4.47 (1H, brs), 7.15 (1H, brt, *J* = 7.2 Hz), 7.18 (1H, brs), 7.19 (2H, brd, *J* = 8.1 Hz), 7.27 (2H, brt, *J* = 7.4 Hz); 13 C NMR (151 MHz, DMSO-d6, the minor rotamer’s signals are marked with an asterisk): δ24.05, 24.39*, 26.00, 26.17*, 27.60*, 27.79, 28.68, 30.15*, 37.54, 38.13*, 39.91, 42.99, 51.01, 52.07, 53.90*, 54.49, 61.48, 61.89*, 71.68, 125.68, 126.51, 128.14, 147.34, 155.27*, 156.08; ESI/APCI MS (m/z): [M + H] + calcd. for C21H33N2O5S, 424.6; found, 425.2; analysis (calcd., found for C21H32N2O5S): C (59.41, 59.45), H (7.60, 7.59), N (6.60, 6.55).

The sample used for structural biology and pharmacology studies was assessed to be >96% pure by LC-MS.

### Cloning and expression of the human OX_2_R-mG_sqiN_ complex

The cloning, expression, purification, cryo-EM data collection, and structure determination for the two OX_2_R-G protein complexes described in this paper were carried out independently. For the OX_2_R-mG_sqiN_ complex the receptor construct contained residues 1–406 of wild-type human OX_2_R (OX_2_R^1–406^). The C-terminal residues 407-444 of OX_2_R were removed to confer slightly improved expression and purification for this complex. Compared to wild-type OX_2_R, OX_2_R^1–406^ displayed similar potencies for activation by TAK-925 and OxB (Supplementary Fig. 11, Supplementary Table 2). The resulting construct was cloned into a modified pFastBac (ThermoFisher) baculovirus expression vector with the HA signal sequence followed by a FLAG tag at the N-terminus. We were unable to isolate a stable complex between OX_2_R and full-length wild-type G_αq_ using the co-expression and purification strategy described below. Instead, an engineered G_αq_ construct called mG_αsqiN_ was made by modifying the chimeric G_α_ protein ‘mini-G_s/q_71’ previously described^26^. Briefly, the linker GGSGGSGG was deleted and residues 1–27 at the N terminus was replaced by residues 1–30 of G_αi1_. A second pFastBac baculovirus was made with this mini-G_αqiN_ gene. An additional pFastBac-Dual baculovirus was made with human G_β1_ and human G_λ2_ genes. An 8xHis tag was placed at the N-terminus of G_λ2_. OX_2_R^1–406^, mG_αsqiN_, G_β1_, and His_8_-tagged G_λ2_ were co-expressed in *Spodoptera frugiperda* (Sf9) cells with the addition of all three baculoviruses (ratio of 1 OX_2_R^1–406^:1 mG_αsqiN_:1G_β1_G _λ2_) to Sf9 cells at a density of 3 × 10^6^ per ml, along with 1 μM TAK-925 added to the media during growth. After 48 h, cells were harvested and stored at −80 °C.

### Cloning and expression of the human OX_2_R-G_i1_-scFv16 complex

The coding sequence of wild type human OX_2_R (residues 2–444) was synthesized and sub-cloned into pFastbac with an N-terminal FLAG tag followed by a fragment of β_2_AR N-terminal 1-24aa before OX_2_R, and a C-terminal 2xMBP-His_8_ tag after OX_2_R. A TEV cleavage site was inserted between OX_2_R and the MBP tag. The prolactin precursor sequence was used as a signal peptide to increase protein expression. A dominant-negative bovine G_αi1_ construct called DNG_αi1_ was generated by site-directed mutagenesis to incorporate mutations G203A^51^ and A326S^52^ to decrease the affinity of nucleotide binding and increase the stability of the G_αβγ_ complex. All three G protein complex components, human G_αi1_, human G_β1_ and human G_γ2_, were cloned into pFastbac individually. For OX_2_R-G_i1_, scFv16 was cloned into pFastbac with a GP67 signal peptide at the N-terminal and a TEV cleavage-His_8_ tag at the C-terminus. Baculoviruses for OX_2_R, DNG_αi1_, His_8_-tagged G_β1_, G_γ2_, and scFv16 were co-expressed in Sf9 cells. Cell cultures were grown to a density of 3.5 × 10^6^ cells/mL and infected with all five baculoviruses at a ratio of 1:1:1:1:1. 48 h after infection, cells were harvested and stored at −80 °C for further use.

### Cloning, expression and purification of scFv16

For OX_2_R-mG_sqiN_, scFv16 was expressed and purified separately. A synthesized DNA fragment encoding scFv16^25^ was cloned into pFastBac, with a melittin signal sequence at the N-terminus and a 10xHis tag at the C-terminus. The resulting construct was expressed in Sf9 cells. scFv16 was purified as previously described^53^. In brief, secreted scFv16 in the cell culture media was separated from Sf9 cells by centrifugation, and 10 mM Tris buffer pH 8.0 was added to balance pH. Then 1 mM Ni_2_SO_4_ and 5 mM CaCl_2_ were added to quench chelating agents. The media containing scFv16 was loaded onto Ni-NTA affinity resin by gravity, and the column was washed with 20 column volumes of buffer consisting of 50 mM HEPES pH 7.4, 150 mM NaCl and 10 mM imidazole. Protein was eluted from Ni-NTA resin with 10 column volumes of buffer consisting of 50 mM HEPES, 150 mM NaCl and 250 mM imidazole. The eluate was concentrated and run on a Superdex 200 gel filtration column. The peak corresponding to monomeric scFv16 was collected, concentrated and frozen until further use.

### Purification of the human OX_2_R-mG_sqiN_-scFv16 complex

Cell pellets from 6L culture of the expressed OX2R-G_q_ complex were resuspended and lysed for 30 min at 4 °C in a hypotonic buffer consisting of 10 mM HEPES pH 7.4, 160 μg/ml benzamidine, 100 μg/ml leupeptin,1 mM MgCl_2_, 0.1 mM TCEP, Apyrase (0.5 mU/ml, NEB) and 1 μM TAK-925. Lysed cells were spun down and homogenized using a dounce tissue grinder (Wheaton) in buffer consisting of 50 mM HEPES pH 7.4, 150 mM NaCl, 160 μg/ml benzamidine, 100 μg/ml leupeptin, 1 mM MgCl_2_, 1% (w/v) lauryl maltose neopentyl glycol (LMNG, Anatrace), 0.1% Na Cholate, 0.1% cholesteryl hemi-succinate (CHS, Steraloids), 10% glycerol, 0.5 mM TCEP, Apyrase (15 mU/ml, NEB) and 10 μM TAK-925. Solubilization proceeded for 1 hr at 4 °C, followed by centrifugation at 100,000 × *g* for 30 min at 4 °C. The supernatant was incubated with M1 anti-Flag affinity beads (Sigma) in batch-binding mode overnight at 4 °C in the presence of 2 mM CaCl_2_. After binding, the M1 beads were spun down and washed with 10 column volumes of buffer consisting of 50 mM HEPES pH 7.4, 150 mM NaCl, 0.05% LMNG, 0.005% Na Cholate, 0.005% CHS, 5% glycerol, 1 mM MgCl_2_, 2 mM CaCl_2_, 0.5 mM TCEP and 10 μM TAK-925. The complex was eluted from M1 beads with buffer consisting of 50 mM HEPES pH 7.4, 150 mM NaCl, 0.05% LMNG, 0.005% Na Cholate, 0.005% CHS, 5% glycerol, 0.5 mM TCEP, 10 μM TAK-925, 200 μg/ml FLAG and 5 mM EDTA. The eluted OX_2_R-G_q_ complex was mixed with scFv16 at a molar ratio 1:1.2 and incubated for 30 min on ice. Finally, the mixture was concentrated and applied onto Superose 6 Increase 10/300 size exclusion column (GE Healthcare). The total yield of the complex was ~3 mg and the peak corresponding to the OX_2_R-mG_sqiN_-scFv16 complex was collected, concentrated and used in the following cryo-EM experiments (Supplementary Fig. 1a).

### Purification of the human OX2R-G_i1_-scFv16 complex

Cell pellets from 10L culture were thawed at room temperature and suspended in 20 mM HEPES pH 7.2, 50 mM NaCl, 5 mM MgCl_2_. Complex was formed on membranes with addition of 10 μM TAK-925 and Apyrase (25 mU/mL, NEB), and incubation for 1.5 h at room temperature. Cell membranes were collected by ultra-centrifugation at 100,000 × *g* for 35 min. The membranes were then re-suspended and solubilized in buffer containing 20 mM HEPES, pH 7.2, 100 mM NaCl, 10% glycerol, 0.5% (w/v) n-Dodecyl β-D-maltoside (DDM, Anatrace), 0.1% (w/v) cholesteryl hemisuccinate TRIS salt (CHS, Anatrace), 0.1%(w/v) digitonin (Sigma) for 3 h at 4 °C. The supernatant was isolated after centrifugation at 100,000 × *g* for 45 min and then incubated overnight at 4 °C with pre-equilibrated Flag G1 resin (Genscript). After batch binding, the resin with immobilized protein complex was manually loaded onto a gravity column and washed with 10 column volumes of 20 mM HEPES, pH 7.2, 100 mM NaCl, 0.1% digitonin (w/v), 10 μM TAK-925. Protein was treated with TEV protease and eluted with the same buffer supplemented with 200 μg/ml flag peptide. Elution was concentrated and loaded onto a Superdex 200 10/300 GL increase column (GE Healthcare) pre-equilibrated with buffer containing 20 mM HEPES, pH 7.2, 100 mM NaCl, 0.075% digitonin, and 10 μM TAK-925. The total yield of the complex was ~5 mg and the eluted fractions of monomeric complex were collected and concentrated for cryo-EM experiments (Supplementary Fig. 1b). Note that a different detergent was used for this complex compared to OX_2_R-mG_sqiN_-scFv16 (digitonin versus LMNG) because these purifications were developed independently, and not due to any specific preference of each complex for a different detergent micelle.

### Cryo-EM data acquisition for the OX_2_R-mG_sqiN_-scFv16 complex

Prior to freezing grids, the OX2R-mG_sqiN_-scFv16 complex was concentrated to 9.5 mg/ml. Cryo-EM grids were prepared by applying 3.5 μl of this sample to glow-discharged Quantifoil R1.2/1.3 300-mesh Au holey carbon grids (Quantifoil Micro Tools GmbH, Germany), blotted for 4.5 s under 100% humidity at 4 °C and plunge frozen in liquid ethane cooled by liquid nitrogen using a Mark IV Vitrobot. SerialEM software was used for automated data collection. Images were recorded on a Titan Krios microscope (FEI) operated at 300 kV with a K3 direct electron detector (Gatan) in super-resolution correlated-double sampling counting mode using a slit width of 20 eV on a GIF-Quantum energy filter (Supplementary Fig. 12a). Images were recorded at a nominal magnification of ×81,000, corresponding to a pixel size of 1.08 Å, and a target defocus range from −1.6 to −2.6 μm. Each movie stack was dose-fractionated over 32 frames for a total of 9 s under a dose rate of 8 e^−^/pixel/sec, resulting in a total dose of ~ 64 e^−^/ Å^2^.

### Cryo-EM data acquisition for the OX_2_R-G_i1_-scFv complex

For cryo-EM grid preparation, 3 μl purified OX_2_R-G_i1_-scFv16 complex at a concentration of 8 mg/mL was applied to an EM grid (Quantifoil, 300 mesh Au R1.2/1.3, glow discharged for 30 sec using a Solarus II plasma cleaner (Gatan)) in a Vitrobot chamber (FEI Vitrobot Mark IV). Protein concentration was determined by absorbance at 280 nm using a Nanodrop 2000 Spectrophotometer (Thermo Fisher Scientific). The Vitrobot chamber was set to 95% humidity at 4 °C. The sample was blotted for 2 s before plunge-freezing into liquid ethane. Cryo-EM movie stacks were collected on a Titan Krios microscope operated at 300 kV under EFTEM mode (Supplementary Fig. 12b). Nanoprobe with 1 μm illumination area was used. Data were recorded on a post-GIF Gatan K2 summit camera at a nominal magnification of 130,000, using counting mode. Bioquantum energy filter was operated in the zero-energy-loss mode with an energy slit width of 20 eV. Data collection were performed using Leginon with one exposure per hole. The dose rate was ~8.4 e^−^/Å^2^/sec. The total accumulative electron dose was ~50 e^−^/Å^2^ fractioned over 40 subframes with a total exposure time of 6 s. The target defocus range was set to −1.3 to −1.9 μm.

### Image processing of the OX_2_R-mG_sqiN_-scFv complex

Data was processed in Relion 3.0^54^ following the same general protocol as previously described^53^. Dose-fractioned images were gain normalized, 2×2 Fourier binned, motion corrected, dose-weighted, and summed using MotionCor2^55^. Contrast transfer function parameters were estimated using GCTF^56^. Approximately one thousand particles were picked manually and subjected to 2D classification. Representative projections of the complex were selected as templates for automated particle picking from all images. 8,929,023 extracted particles were 4×4 binned and subjected to 2D classification. A total of 2,661,363 particles were finally selected for 3D classification using the initial model generated by Relion as a reference. After 3 rounds of 3D classification, the classes showing good secondary structure features were selected, combined and re-extracted at the original pixel size of 1.08 Å. After 3D refinement and postprocessing, the resulting 3D reconstitution from 155,322 particles yielded a map at 3.3 Å resolution using the gold-standard FSC criterion (cryo-EM data-processing flowchart is shown in External Data Fig. 2). Local resolution map was calculated using Relion 3.0.

### Image processing of the OX2R-G_i1_-scFv complex

A total of 8453 movie stacks were collected. Each movie stack was aligned and dose weighted by 1.04 Å per pixel using MotionCor2^55^. CTF parameters were determined using CTFFIND4^57^. A total of 2,735,296 particles were auto picked using template picker and extracted with a box size of 256×256 pixels in cryoSPARC^58^. All particles were subject to two rounds of reference-free 2D classification. The following 2D classifications, 3D classifications and refinements were all performed in cryoSPARC. 677,124 particles were selected after two rounds of 2D classification based on the presence of intact complex. This particle set was used to do Ab-Initio reconstruction in four classes, which were then used as 3D volume templates for heterogeneous refinement. 332,173 particles were used for final non-uniform refinement. The global resolution of the final reconstruction is 3.13 Å (cryo-EM data-processing flowchart is shown in External Data Fig. 3). Local resolution map was calculated using cryoSPARC. Surface coloring of the density map was performed using UCSF Chimera^59^.

### Model building and refinement of the OX2R-mG_sqiN_-scFv complex

The active structure of μOR (PDB: 6DDF) as well as structures of mini-G_αq_ (PDB: 6WHA), G_β1λ2_ and scFv16 (PDB: 6VMS) were used as initial models for model rebuilding and refinement. A polyalaline model was made from the μOR structure, and together with structures of mini-G_αq_, G_β1λ2_, and scFv16, components were docked into the cryo-EM density map in Chimera. The resulting model was subjected to autobuilding in Buccaneer^60^, iterative building in Coot^61^ and refinement in Phenix.real_space_refine^62^. Initial coordinates and refinement parameters for the ligand TAK-925 were prepared with DRG web server (http://davapc1.bioch.dundee.ac.uk/cgi-bin/prodrg). MolProbity was used to evaluate the final structures. In the Ramachandran plot, 97.6% and 2.4% of residues were in favored and allowed regions, respectively. Statistics for data collection and refinement are included in Supplementary Table 1. The cryo-EM density map has been deposited in the Electron Microscopy Data Bank under accession code EMD-25399. Atomic coordinates have been deposited in the PDB under accession code 7SR8. Structural figures were made using Pymol (Version 2.0 Schrödinger, LLC) and UCSF Chimera.

### Model building and refinement of the OX2R-G_i1_ complex

The structures of OX_2_R (PDB:5WQC) and G_i_-scFv16 (PDB: 6DDE) were individually placed and rigid-body fitted into the cryo-EM map using Chimera. Manual inspection of the model was performed in Coot^61^, interspersed with restrained real-space refinement using Phenix.real_space_refine^62^. The ligand was placed by hand and then refined in Phenix. All regions of the model were checked thoroughly for registration correctness and fit to density during refinement. Statistics for data collection and refinement are included in Supplementary Table 1. The cryo-EM density map has been deposited in the Electron Microscopy Data Bank under accession code EMD-25389. Atomic coordinates have been deposited in the PDB under accession code 7SQO. Structural figures were made using Pymol and UCSF Chimera.

### Quantum chemistry calculation

Northwest Computational Chemistry Package (NWChem) 6.8.1^63^ was used to perform all electronic structure calculations. All geometries were optimized at the B3LYP/6-31G* level of the *Density-functional* theory (DFT) in gas phase first and then with the solvation model based on density (SMD) model^64^ in aqueous solution. Vibrational frequencies were computed in each case to confirm that each structure was a local minimum on the potential energy surface and to compute thermodynamic quantities. The standard Eckart projection algorithm, as implemented in NWChem, was applied to project out the translations and rotations of the nuclear hessian. Based on the frequencies obtained from the projected hessian, the zero point energy for the molecular system was calculated.

### Inositol phosphate accumulation assay for G_q_ signaling

The IP assay was adapted from a prior precedent^65^. HEK293 cells were maintained in DMEM-high glucose (Millipore Sigma) supplemented with 5% fetal bovine serum (Corning) and penicillin-streptomycin (Millipore Sigma). Receptor constructs used were the full-length wild-type (or mutant) human OX_1_R and OX_2_R sequences cloned into pCDNA3. To enhance the E_max_ in the IP assay, a plasmid encoding full-length human G_αq_ subunit^66^ was co-transfected into HEK293 cells with plasmids encoding orexin receptor constructs using Lipofectamine 3000 (Thermo Fisher). 24 h after transfection, the cells were plated on poly-D-lysine coated (Millipore Sigma) tissue culture-treated 96 well white plates with clear bottoms (Perkin Elmer) at 30,000 cells per well in Inositol-free DMEM (MP Biomedicals) supplemented with 5% fetal bovine serum, 4 mM L-glutamine (Millipore Sigma), penicillin-streptomycin, and about 5 uCi (5 ul) per ml myo-[2-3H] inositol (Perkin Elmer). The following day, the media was replaced with agonists that were diluted in HBSS (Millipore Sigma) containing 10 mM lithium chloride (Millipore Sigma) and incubated at 37 °C for 45 min. Then agonists were removed and cells were lysed on ice for 30 min with 10 mM formic acid (Millipore Sigma). 1.25 mg of poly-lysine coated YSI SPA beads (Perkin Elmer) was added to each well. The plates were mixed on an orbital shaker for 30 min. The plates were read on a MicroBeta scintillation counter (Perkin Elmer) the following day. IP accumulation assays on different constructs were performed in *n* ≥ 3 independent experiments (each done in duplicate), where *n* is shown in Supplementary Table 2. Cpm data were normalized to OX_2_R WT E_max_, and dose responses were fitted to the three-parameter model ‘log(agonist) vs response’ in GraphPad Prism 9 (GraphPad Software). Fitted pEC_50_ values were analyzed for statistical significance compared to OX_2_R WT (with TAK-925 or orexin B) using one-way ANOVA followed by Dunnett’s test. All pharmacological parameters are displayed in Supplementary Table 2.

### β-arrestin PathHunter assay

β-Arrestin recruitment activity was measured by using the PathHunter system (DiscoveRX) according to the manufacturer’s instruction. Receptor constructs used were the full-length wild-type (or mutant) human OX_2_R sequences cloned into pCMV-ProLink (DiscoveRX). CHO-K1 cells stably expressing EA β-arrestin2 (DiscoveRX) were plated on 384 well white plates (Thermo Fisher) at 4000 cells/well in HamF12 (Wako Pure Chemical Industries) supplemented with 10% fetal bovine serum (Corning) and penicillin-streptomycin (Wako Pure Chemical Industries) and incubated overnight at 37 °C under 5% CO_2_. Cells were transfected with plasmids encoding wild type or each mutant ProLink-tagged human OX_2_R using FuGENE HD (Promega). The following day, the media was replaced with HBSS (GIBCO) containing 20 mM HEPES (GIBCO) and 0.1% BSA (Wako Pure Chemical Industries), and treated with TAK-925 for 2 h at 37 °C. Detection reagent was added and incubated for 1 h at room temperature. Luminescent signal was detected using an EnVision plate reader (PerkinElmer). β-arrestin assays on different constructs were performed in two or three independent experiments (each done in quadruplicate). Dose response curves (shown in Supplementary Fig. 8) were fitted to the three-parameter model ‘log(agonist) vs response’ in GraphPad Prism to determine pEC50 values. Fitted pEC_50_ values were analyzed for statistical significance compared to OX_2_R WT (with TAK-925 or orexin B) using one-way ANOVA followed by Dunnett’s test.

### Inhibition of forskolin-stimulated cAMP production

To measure G_i_ signaling^67^_,_ HEK293 cells were transfected with pGloSensor™−22F cAMP Plasmid (Promega) and CMV expression plasmid encoding human OX_2_R. To enhance the signal for the cAMP assay, we co-transfected a G_i_ expression plasmid (CMV-driven) in which the full-length human G_αi1_ subunit sequence was cloned into pcDNA3. The following day, cells were harvested and washed with assay buffer, HBSS with 20 mM HEPES pH 7.4 (Millipore Sigma). Cells were suspended in assay buffer containing 0.5 mg/ml Luciferin (Gold Biotechnology) and plated on 96 well tissue culture-treated white plates with opaque bottoms (Thermo Fisher). The cells were incubated at 37 °C for 90 min. Forskolin (Millipore Sigma) diluted in assay buffer was added to wells for a final concentration of 5 mM. Luminescence was read at room temperature repeatedly in a CLARIOstar microplate reader (BMG Labtech) until the signal stopped increasing. Agonist diluted in assay buffer was added from a 7X stock and again the plates were read repeatedly until the signal was stable. Assays for either TAK-925 or orexin B were performed in 3 independent experiments (each done in duplicate). cAMP luminescence data were plotted as a fraction of the maximal signal with forskolin, and dose responses were fitted to the three-parameter model ‘log(inhibitor) vs response’ in GraphPad Prism 9 (GraphPad Software). Both TAK-925 nor orexin B (Fig. 5d) displayed minimal diminution of cAMP signal comparable to cells without transfected OX_2_R. As a positive control, cells were transfected with pGloSensor™−22F cAMP, D2 dopamine receptor, and G_αi1_ plasmids, and tested for their response to dopamine (Millipore Sigma) after forskolin treatment. The measured IC_50_ for dopamine was 4.9 ± 1.1 nM.

### Enzyme-linked immunosorbent assay (ELISA) for cell surface expression

The cell surface expression levels of wild-type and mutant human OX_1_R and OX_2_R constructs were quantified by an enzyme-linked immunosorbent assay (ELISA). As above in IP accumulation and cAMP assays, wild-type and mutant human OX_1_R and OX_2_R constructs were cloned into pcDNA3. HEK293 cells were transfected with pCDNA3 expression plasmids encoding receptor constructs and full-length human G_αq_ subunit, using Lipofectamine 3000 (Thermo Fisher). 24 h after transfection, the cells were plated on poly-D-lysine coated (Millipore Sigma) tissue culture-treated 24 well clear plates (Corning) at 200,000 cells per well in DMEM-high glucose (Millipore Sigma) supplemented with 5% fetal bovine serum (Corning) and penicillin-streptomycin (Millipore Sigma). After 24 h at 37 °C w/5% CO_2_, the media was aspirated and cells were washed with 200 μL/well of TBS buffer twice. 400 μL/well of 4% paraformaldehyde (PFA) were then added for fixation of cells, and cells were incubated for 30 min on ice. Cells were washed with 200 μL/well of TBS buffer three times, followed by addition of 1% BSA. Incubation proceeded for 1 h at room temperature. After 1% BSA in TBS was aspirated, 200 μL/well of 9.7 μg/ml of M1-Flag antibody (Sigma) in TBS w/1%BSA was added and cells were incubated at room temperature for 1 h. Cells were then washed with 200 μL/well of TBS w/1%BSA buffer three times, followed by addition of HRP-coupled secondary antibody with 1:2000 dilution in TBS w/1%BSA. Incubation proceeded for 1 h at room temperature. After cells were washed with 200 μL/well of TBS w/1%BSA buffer three times, 200 μL/well of TMB-ELISA (Thermo Fisher) was added. After a short time of incubation, 100 μL/well of colored solution was transferred to 96 well clear plates (Corning) containing 100 μL/well of 1 M H_2_SO_4_ to stop reaction. Absorbance produced by HRP activity was immediately measured at 450 nm using a CLARIOstar microplate reader (BMG LABTECH). After measuring HRP activity, cells were washed with 200 μL/well of TBS twice and incubated with 200 μL/well of 0.2% (w/v) Janus green for 30 min. To remove extra Janus green, cells were washed with water three times, followed by addition of 800 μL/well of 0.5 M HCl. 200 μL/well of colored solution was transferred to the same 96 well clear plates (Corning). Absorbance at 595 nm was recorded by a CLARIOstar microplate reader (BMG LABTECH). For each well condition, the normalized cell surface expression was determined by dividing absorbance at 450 nm by absorbance at 595 nm. Experiments were repeated three times. The data were analyzed by one-way ANOVA (and Nonparametric or Mixed) and plotted as column with scattered points using GraphPad Prism 9 (GraphPad Software).

### Reporting summary

Further information on research design is available in the Nature Research Reporting Summary linked to this article.

## Supplementary information

Supplementary Information

Reporting Summary

## Data Availability

The structural data generated in this study have been deposited in the Protein Data Bank (PDB) under accession codes 7SQO and 7SR8, and cryo-EM maps have been deposited in the Electron Microscopy Data Bank (EMDB) under accession codes EMD-25389 and EMD-25399. The pharmacological data generated in this study are compiled in the Source Data file provided with this paper. Source data are provided with this paper.

## Source data

Source Data
