# Supplementary material for: Molecular mechanism of the wake-promoting agent TAK-925
Source: Nat Commun. 2022 May 25;13:2902. doi: 10.1038/s41467-022-30601-3 (PMC9133036; doi:10.1038/s41467-022-30601-3)
Supplement: Supplementary file 1 — Supplementary Information [file 41467_2022_30601_MOESM1_ESM.pdf]

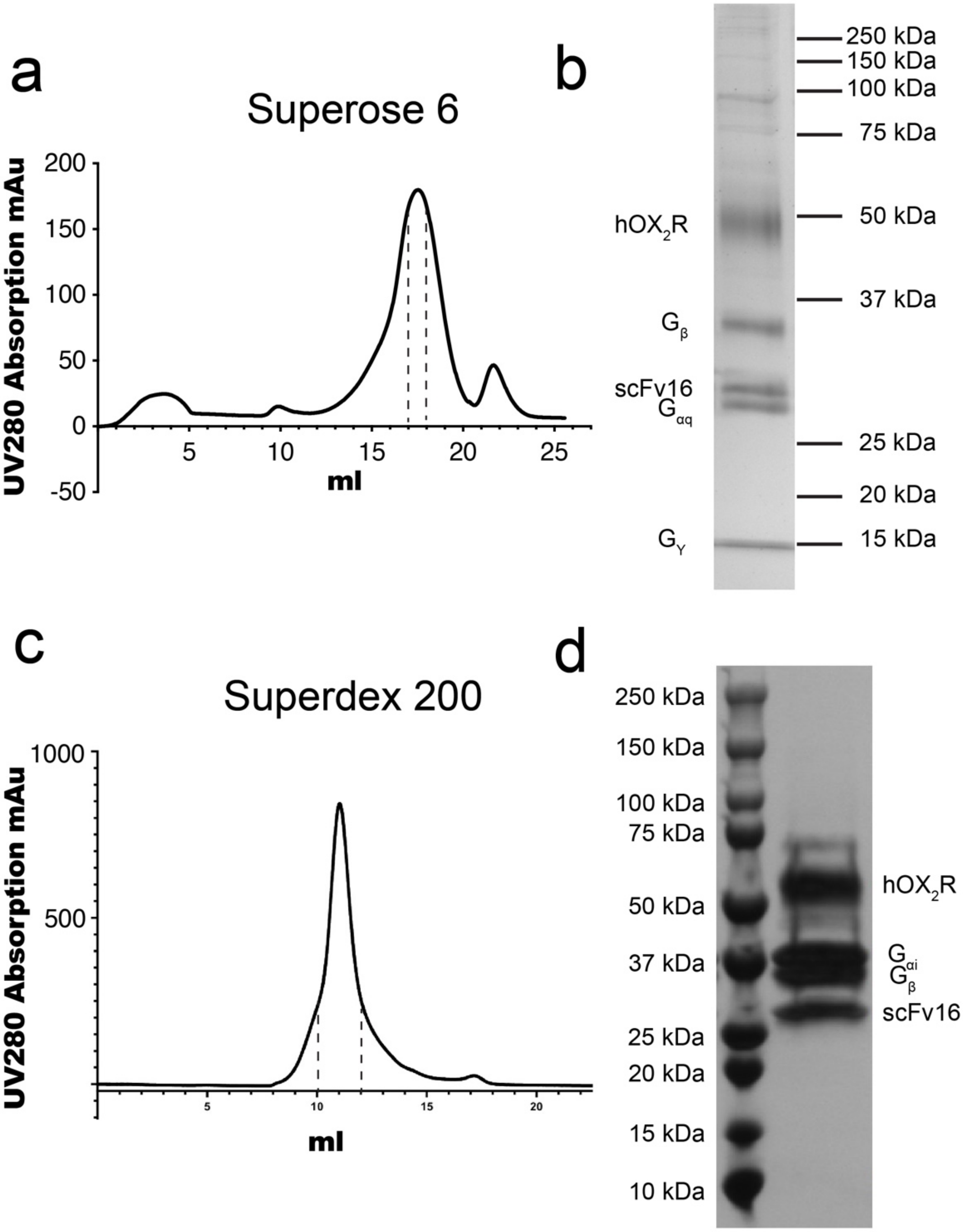

Supplementary Fig. 1. Purification of OX$_2$R-G$_{sqiN}$ and OX$_2$R-G$_{i1}$.

(a) Gel filtration of OX$_2$R-mG$_{sqiN}$-scFv16 purified by M1 FLAG affinity chromatography. Dashed line indicates the peak fractions used for concentration and structure determination.

(b) Coomassie-stained PAGE of the isolated peak of OX$_2$R-mG$_{sqiN}$-scFv16 from gel filtration. This purification was repeated independently 3 times, with similar results.

(c) Gel filtration of OX$_2$R-G$_i$-scFv16 purified by FLAG affinity chromatography. Dashed line indicates the peak fractions used for concentration and structure determination.

(d) Coomassie-stained PAGE of the isolated peak of OX$_2$R-G$_{i1}$-scFv16 from gel filtration. Note that the G$_\gamma$ subunit was not visible by Coomassie staining, likely due to poor staining of the low-MW species, however cryo-EM reconstruction (Figure 1d) showed that this subunit was present in the eluted complex. This purification was repeated independently 3 times, with similar results.

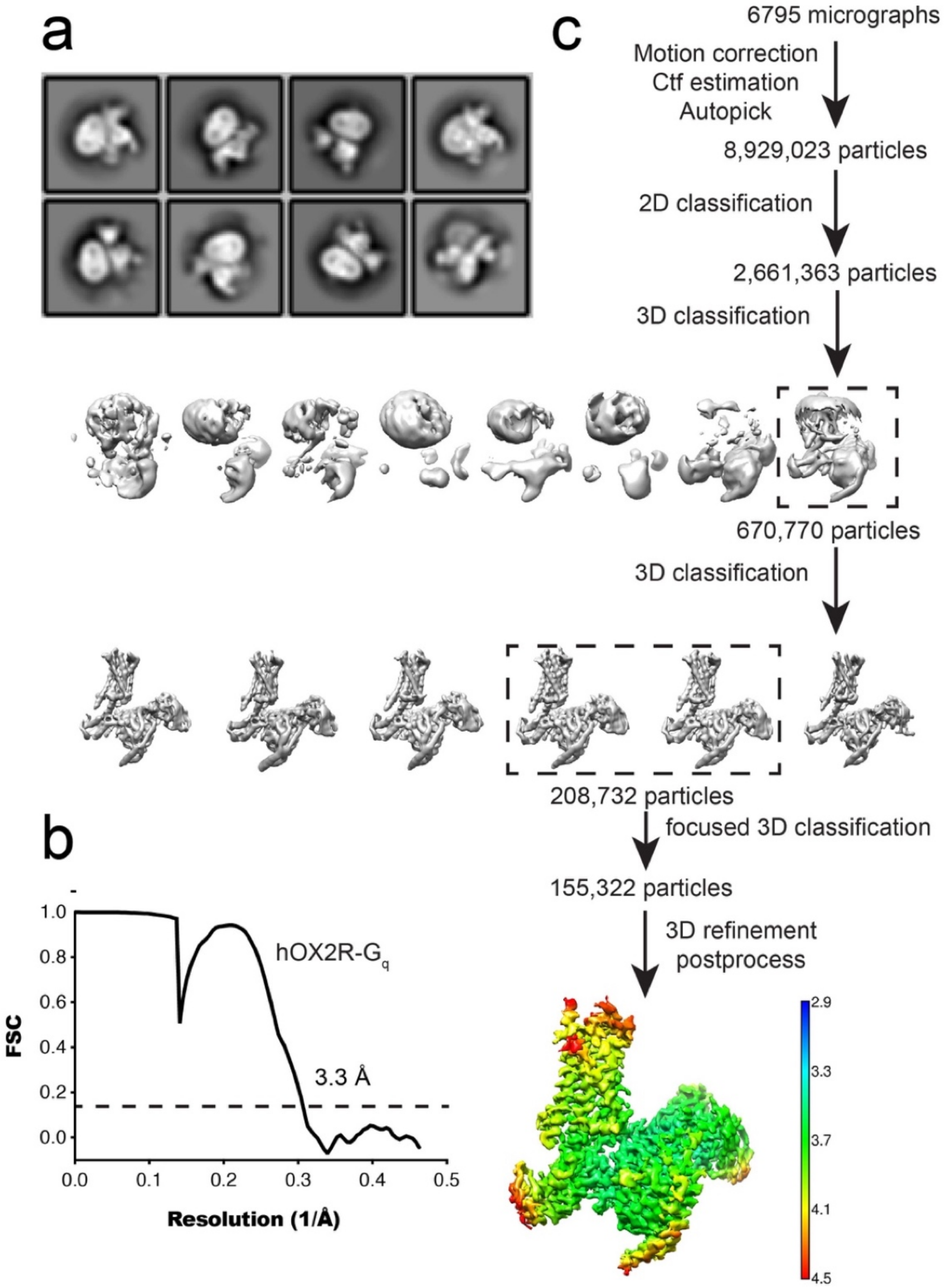

Supplementary Fig. 2. Cryo-EM analysis of $OX_2R\text{-}mG_{sqiN}$.
(a) Representative 2D class averages from Relion.
(b) Gold-standard FSC (Fourier Shell Correlation) curve comparing two halves of the data from the 3D reconstruction, generated during postprocessing in Relion. The dashed line indicates FSC 0.143.
(c) Image processing procedure demonstrating selection of 3D classes in Relion that were used in final 3D reconstruction, along with a final map of the complex colored according to local resolution estimation in Relion and displayed by UCSF Chimera.

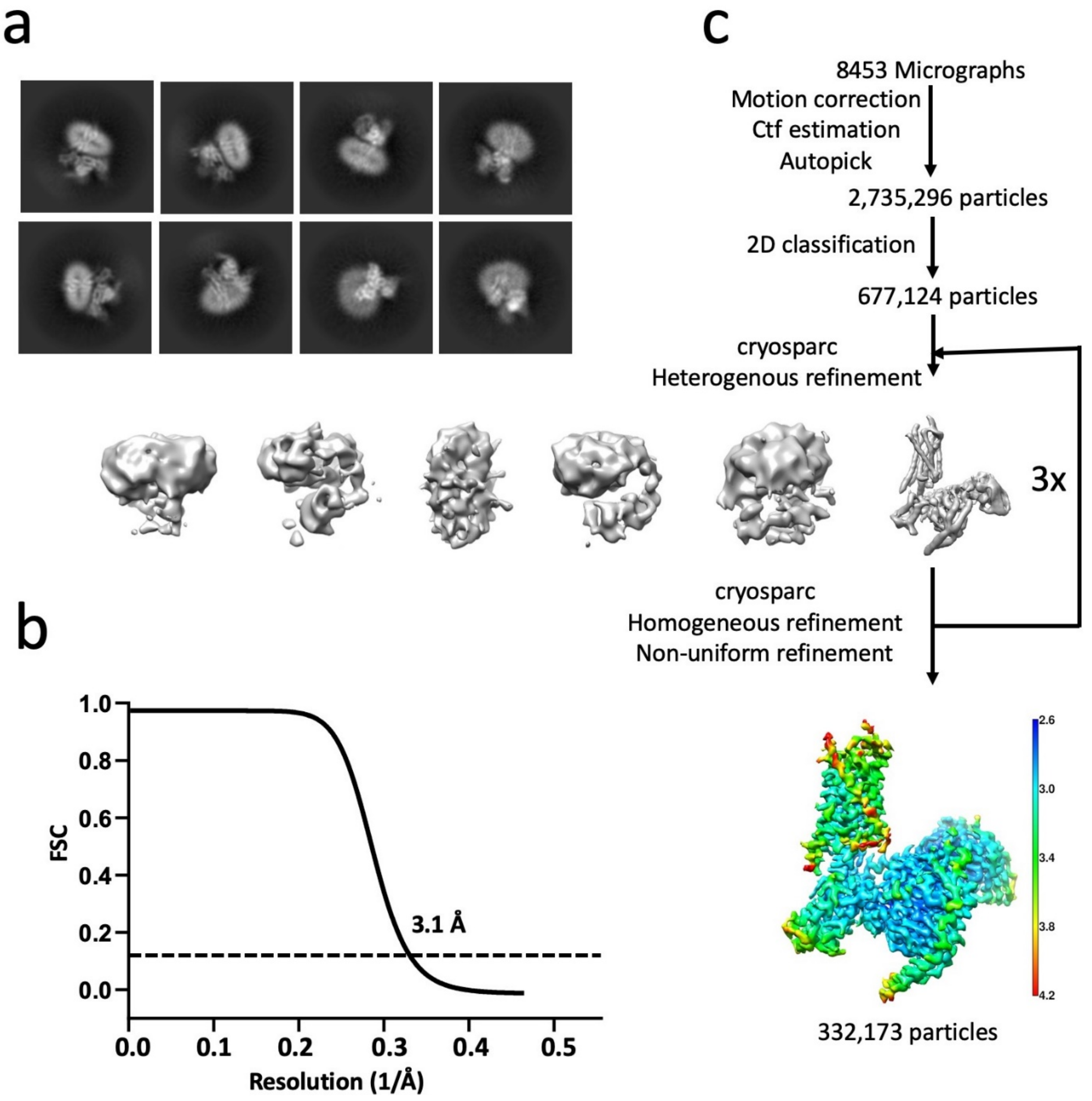

Supplementary Fig. 3. Cryo-EM analysis of OX_2R-G_{i1}.
(a) Representative 2D class averages from cryoSPARC.
(b) Gold-standard FSC (Fourier Shell Correlation) curve comparing two halves of the data from the 3D reconstruction, generated during postprocessing in cryoSPARC. The dashed line indicates FSC 0.143.
(c) Image processing procedure demonstrating selection of particles during heterogeneous refinement in cryoSPARC that were used in final 3D reconstruction, along with a final map of the complex colored according to local resolution estimation in cryoSPARC and displayed by UCSF Chimera.

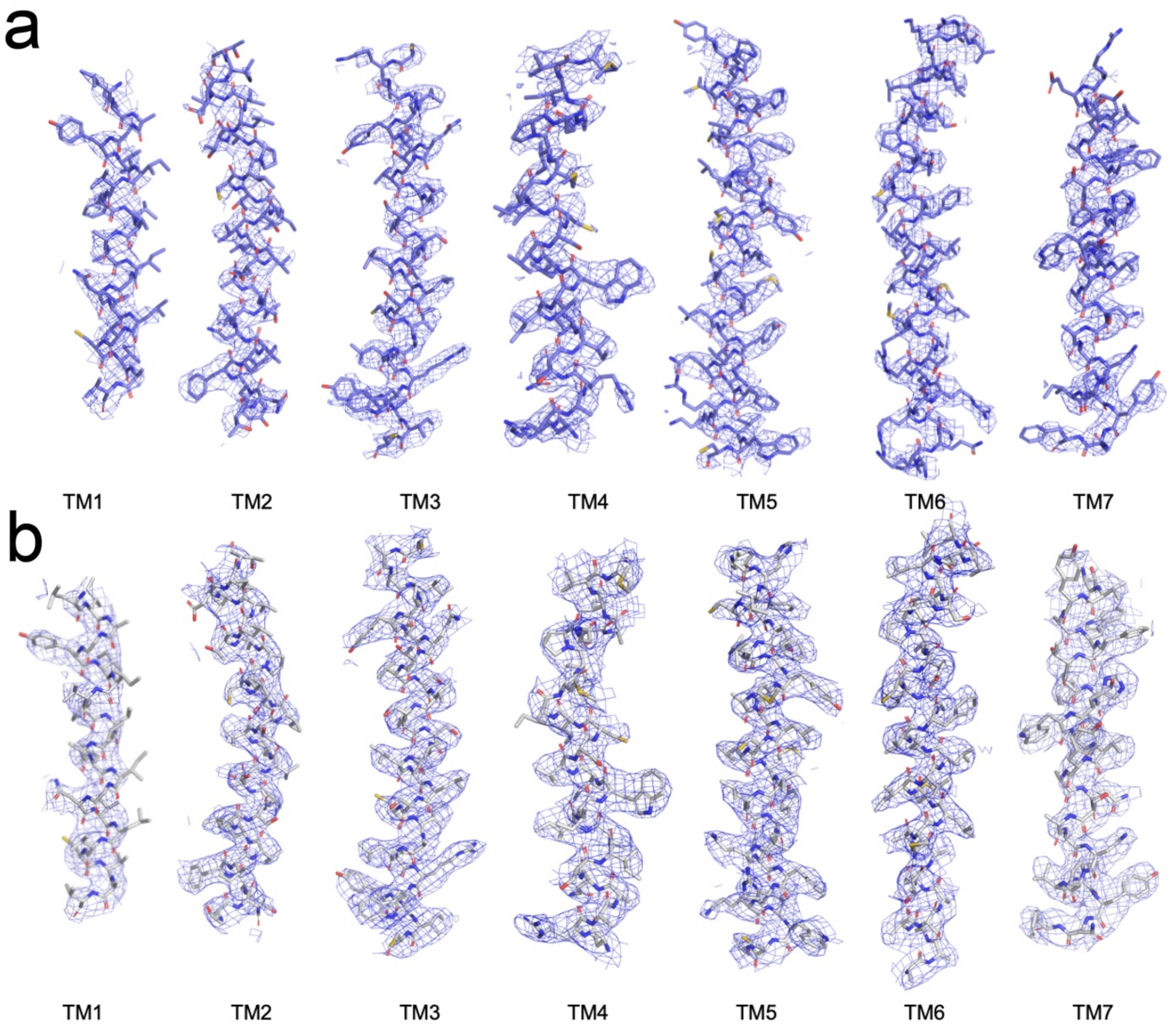

Supplementary Fig. 4. Density for OX_2R in the cryo-EM structures.
(a) Transmembrane helix density in the OX_2R-mG_{sqiN} cryo-EM map. Density map (and model) was displayed by UCSF Chimera with contour level 0.033 (5 sigma).
(b) Transmembrane helix density in the OX_2R-G_i cryo-EM map. Density map (and model) was displayed by UCSF Chimera with contour level 0.5 (5 sigma).

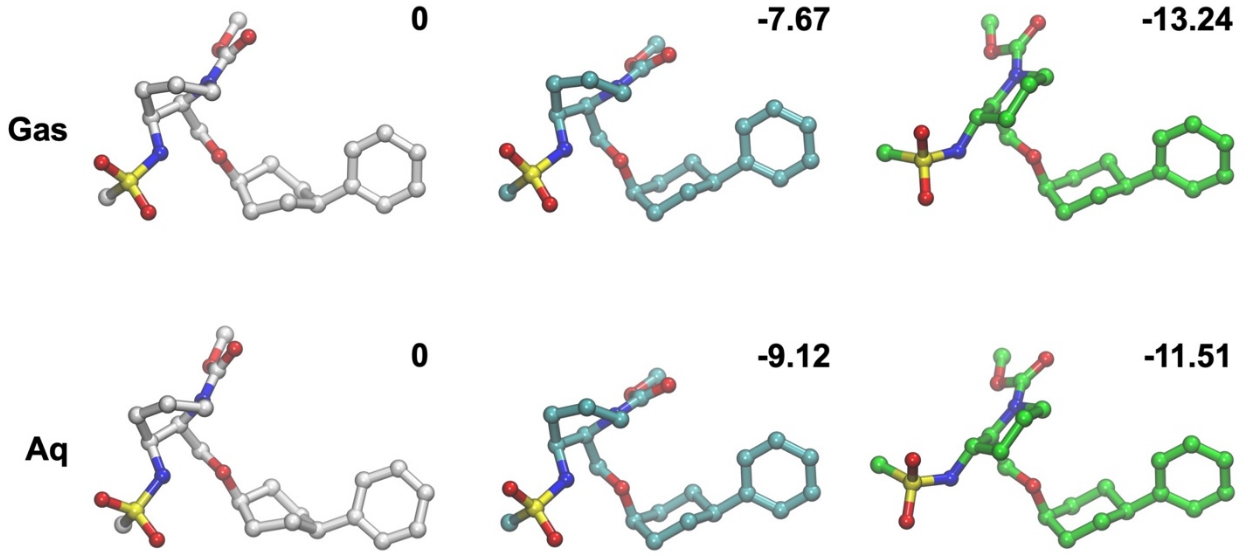

Supplementary Fig. 5. Optimized structures of TAK-925 at DFT B3LYP/6-31G* level in gas phase (top row) and aqueous phase (bottom row). The compound with both piperidine and cyclohexyl rings in skewed-boat conformation are colored in white, the compound with piperidine in skewed-boat and cyclohexyl in chair conformation are colored in cyan, and the compound with both piperidine and cyclohexyl rings in chair conformation are shown in green. The relative free energies of the compound in different conformations are shown in the upper right corner of each molecule. Hydrogen atoms of the compounds are not shown for clarity.

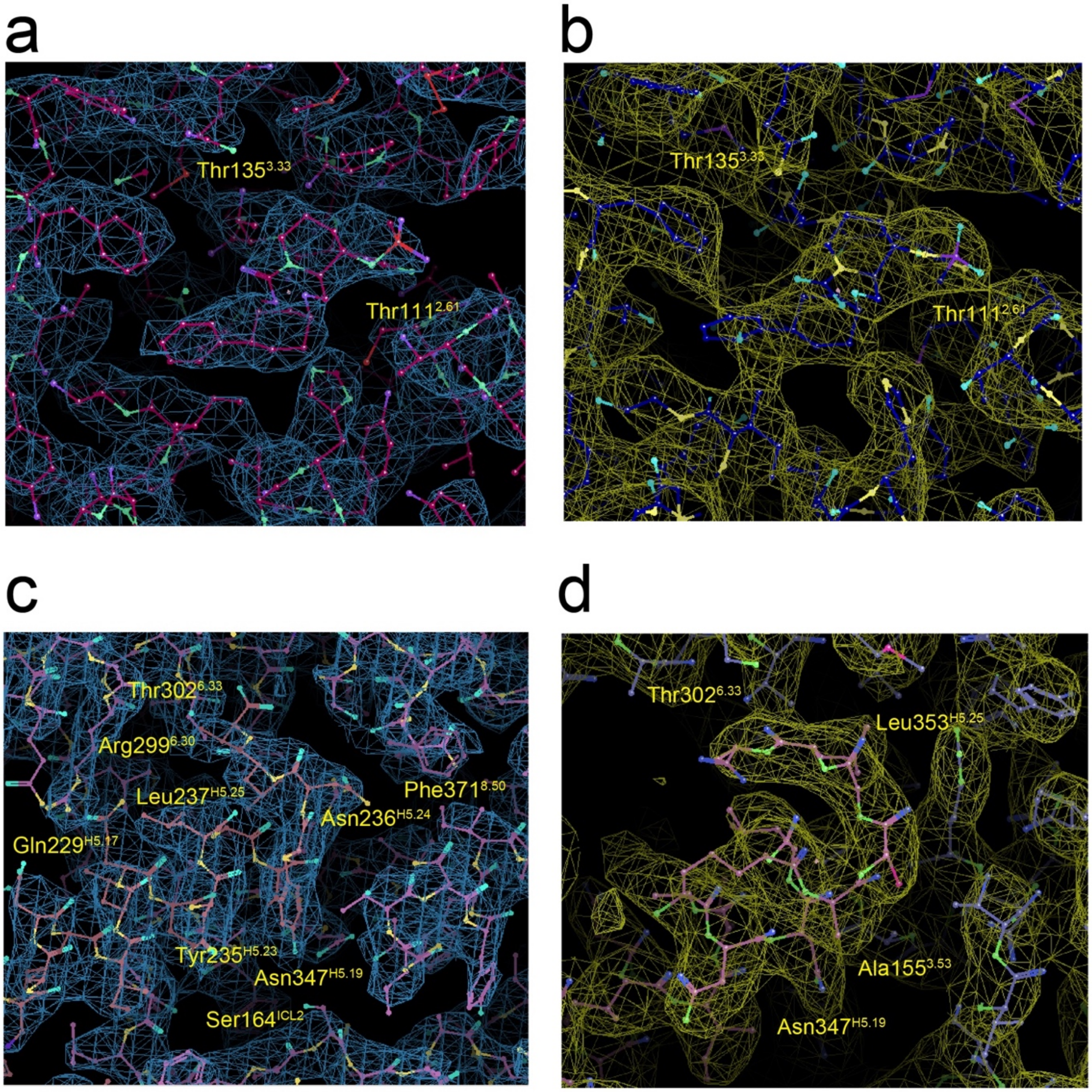

Supplementary Fig. 6. Cryo-EM maps for TAK-925 binding pockets and G protein interfaces.
(a) Region of the density map for the OX$_2$R-mG$_{sqiN}$ complex centered on the TAK-925 binding pocket. The cryo-EM density (blue mesh) was displayed in Coot at sigma level 5. The coordinates are shown as sticks colored by heteroatom with purple carbons, red oxygens, and cyan nitrogens.
(b) Region of the density map for the OX$_2$R-G$_{i1}$ complex centered on the TAK-925 binding pocket. The cryo-EM density (yellow mesh) was displayed in Coot at sigma level 5. The coordinates are shown as sticks colored by heteroatom with blue carbons, cyan oxygens, and yellow nitrogens.
(c) Region of the density map for the OX$_2$R-mG$_{sqiN}$ complex centered on the receptor-G$_\alpha$ interface. The cryo-EM density (blue mesh) was displayed in Coot at sigma level 5. The coordinates are shown as sticks colored by heteroatom with purple carbons for receptor, pink carbons for G$_\alpha$, cyan oxygens, and yellow nitrogens.
(d) Region of the density map for the OX$_2$R-G$_{i1}$ complex centered on the receptor-G$_\alpha$ interface. The cryo-EM density (yellow mesh) was displayed in Coot at sigma level 5. The coordinates are shown as sticks colored by heteroatom with light blue carbons for receptor, pink carbons for G$_\alpha$, blue oxygens, and green nitrogens.

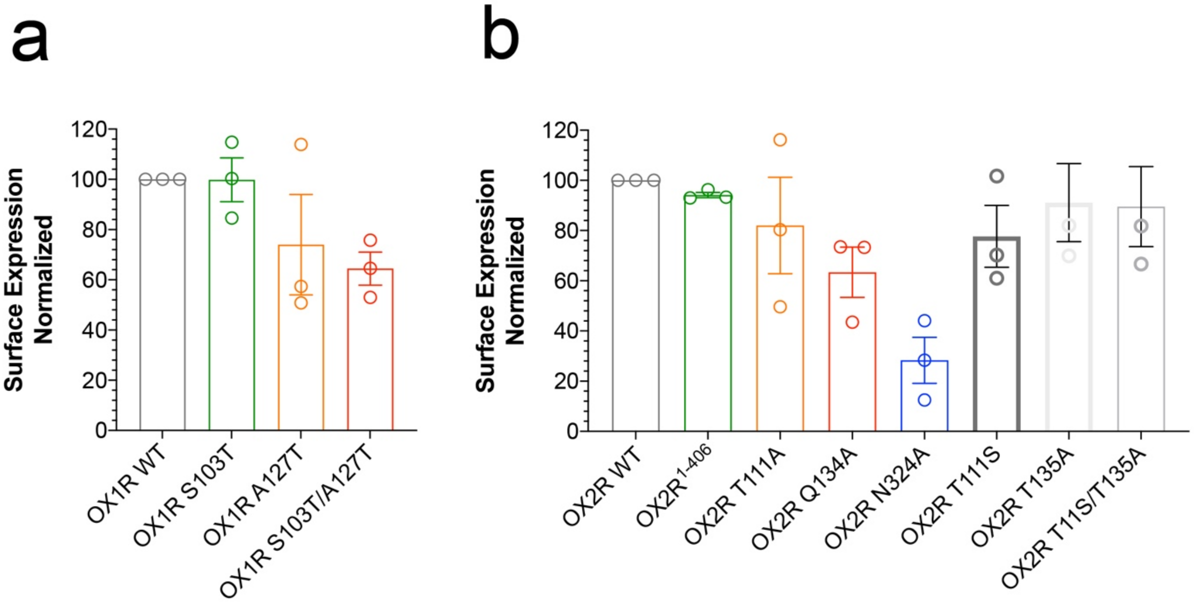

Supplementary Fig. 7. Cell surface expression of receptor constructs.

(a) Cell surface ELISA signals for mutant OX_1R constructs used in Figure 3b. Data points represent the normalized signal from dividing A_{450} (ELISA signal using M1-Flag as primary antibody) by A_{595} (Janus green for total cells). Each column represents n=3 independent experiments with separate transfections. The data were plotted as columns with scattered points using GraphPad Prism (GraphPad Software). Top of each column represents the mean value (centre), while error bars are ± SD.

(b) Cell surface ELISA signals for mutant OX_2R constructs used in this study (Figs. 2d, 3c, and Supplementary Fig. 10). Data points represent the normalized signal from dividing A_{450} (ELISA signal using M1-Flag as primary antibody) by A_{595} (Janus green for total cells). Each column represents n=3 independent experiments with separate transfections. The data were plotted as columns with scattered points using GraphPad Prism (GraphPad Software). Top of each column represents the mean value (centre), while error bars are ± SD.

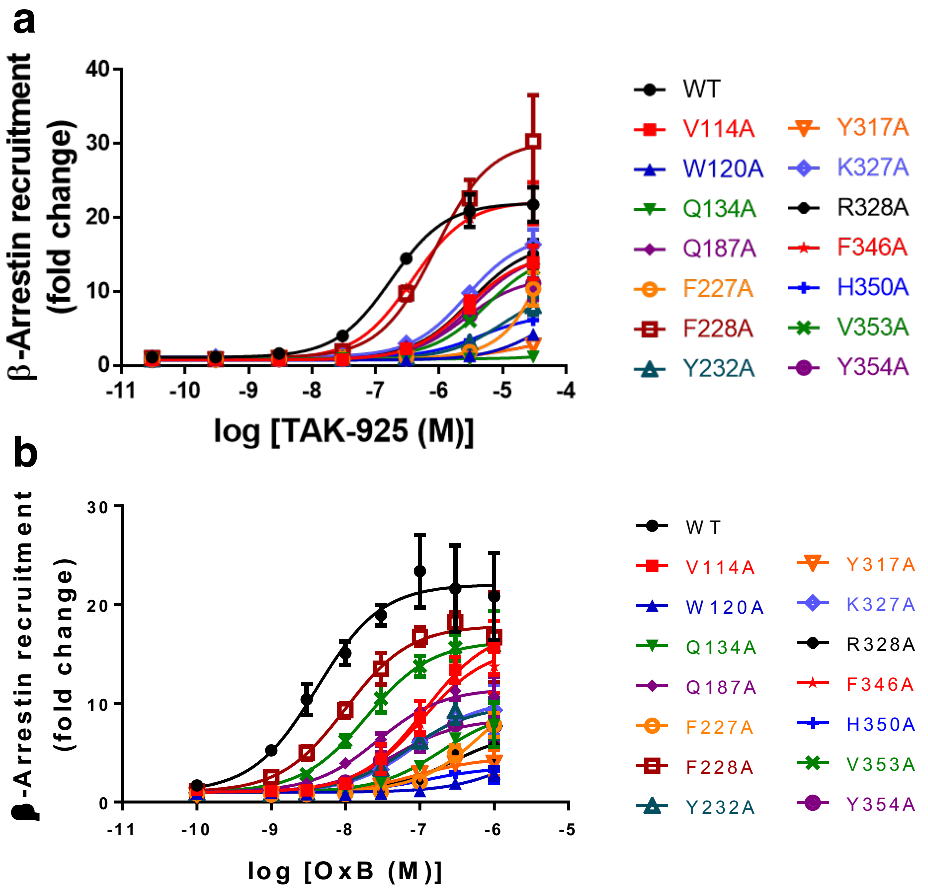

Supplementary Fig. 8. β-arrestin recruitment responses of OX_2R mutants.

(a) Dose responses to TAK-925. Data presented are the means ± SD from n=3 independent experiments, each performed in quadruplicate. Data were fitted to a three-parameter logistic equation using GraphPad Prism to determine pEC_{50} values. Source data are provided as a Source Data file.

(b) Dose responses to orexin B. Data presented are the means ± SD from n=3 independent experiments, each performed in quadruplicate. Data were fitted to a three-parameter logistic equation using GraphPad Prism to determine pEC_{50} values. Source data are provided as a Source Data file.

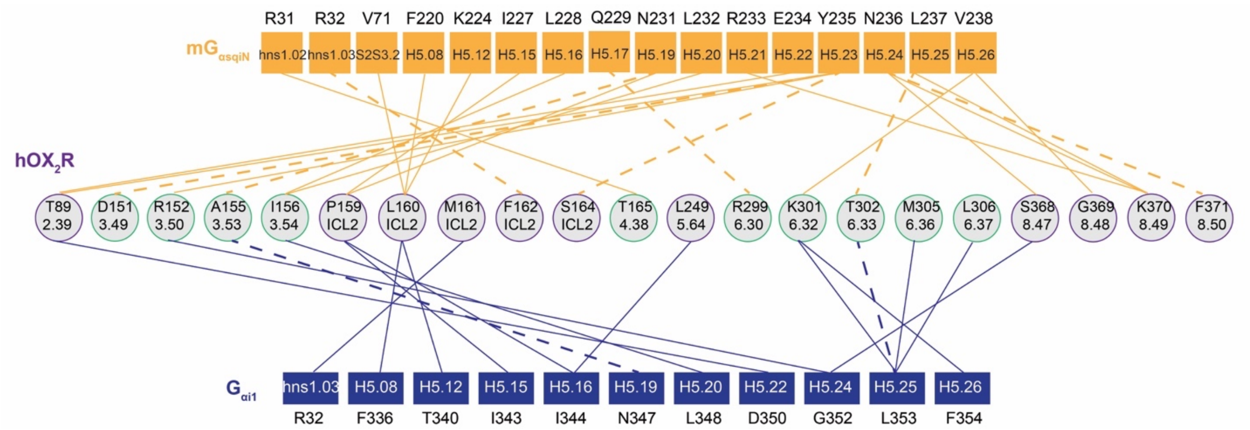

Supplementary Fig. 9. Contact map between OX_2R and $G_{\alpha sqiN}$ or $G_{\alpha i1}$.

Van der waals interactions between OX_2R and $G_{\alpha sqiN}$ are represented by orange solid lines, and interactions between OX_2R and $G_{\alpha i1}$ are represented by blue solid lines. Hydrophobic contacts are defined by distances between residues of less than or equal to 4 Å. Hydrogen bonds between OX_2R and $G_{\alpha sqiN}$ are represented by orange dashed lines, and hydrogen bonds between OX_2R and $G_{\alpha i1}$ are represented by blue dashed lines. Hydrogen bonds are defined by distances between donor and acceptor heteroatoms of less than or equal to 3.3 Å.

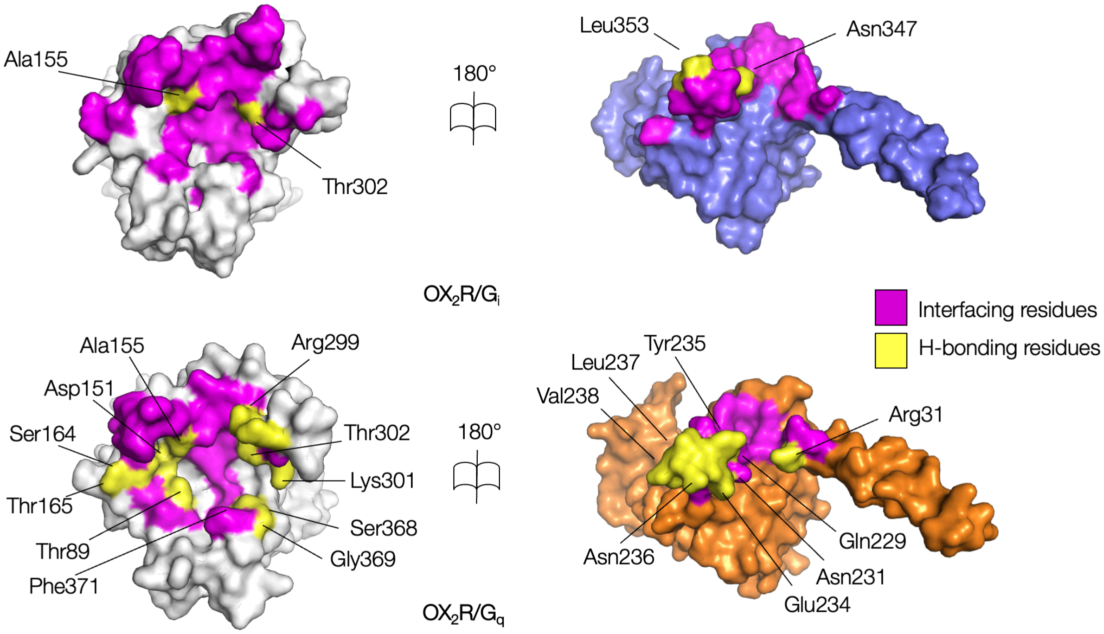

Supplementary Fig. 10. Interfaces between OX_2R and G proteins.
(a) OX_2R-G_{i1} interface. Receptor and G_α subunits are shown in 'open book' format where the two proteins have been rotated away from each other 180°. Structures were displayed as surfaces in Pymol, with receptor surface colored gray, G_α surface colored blue, residues involved in van der Waals contacts colored magenta, and residues involved in H-bonds colored yellow.
(b) OX_2R-mG_{sqiN} interface. Receptor and Gα subunits are shown in 'open book' format where the two proteins have been rotated away from each other 180°. Structures were displayed as surfaces in Pymol, with receptor surface colored gray, G_α surface colored orange, residues involved in van der Waals contacts colored magenta, and residues involved in H-bonds colored yellow.

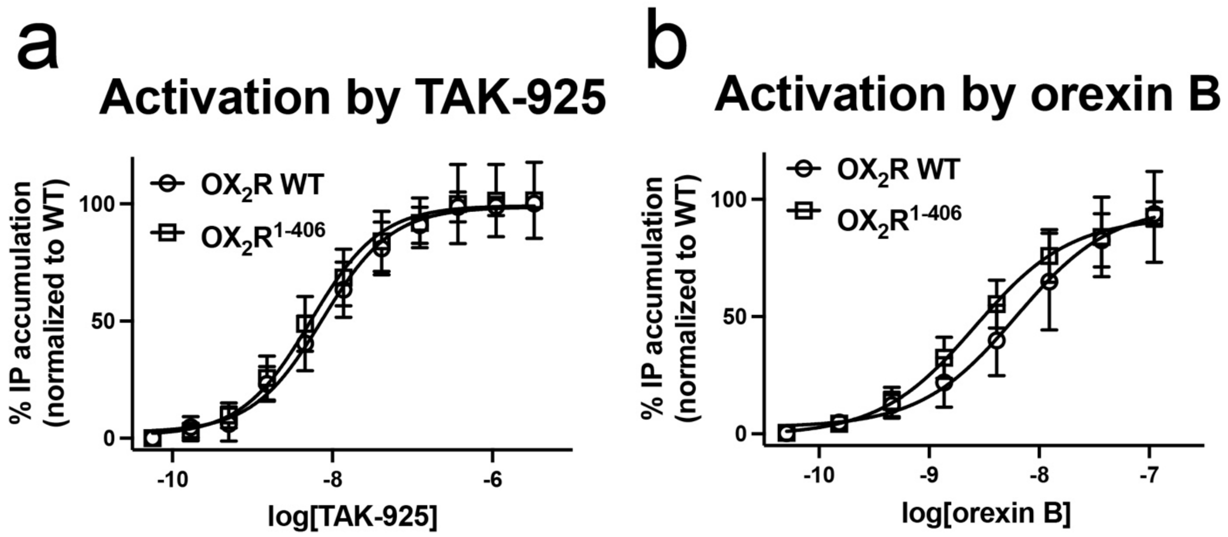

Supplementary Fig. 11. G_q signaling of C-terminally truncated OX_2R versus wild-type OX_2R.
(a) Stimulation of G_q by OX_2R wild-type (WT) versus OX_2R with C-terminal 25 residues removed (OX_2R^{1-406}) in the presence of TAK-925. Data presented are the means from n ≥3 independent experiments (each performed in duplicate), where n is shown in Supplementary Table 2. Error bars are ± SD. Data were normalized to WT and fitted to the three-parameter model 'log(agonist) vs response' in GraphPad Prism 9. Source data are provided as a Source Data file.
(b) Stimulation of G_q by OX_2R wild-type (WT) versus OX_2R with C-terminal 25 residues removed (OX_2R^{1-406}) in the presence of orexin B. Data presented are the means from n ≥3 independent experiments (each performed in duplicate), where n is shown in Supplementary Table 2. Error bars are ± SD. Data were normalized to WT and fitted to the three-parameter model 'log(agonist) vs response' in GraphPad Prism 9. Source data are provided as a Source Data file.

a

b

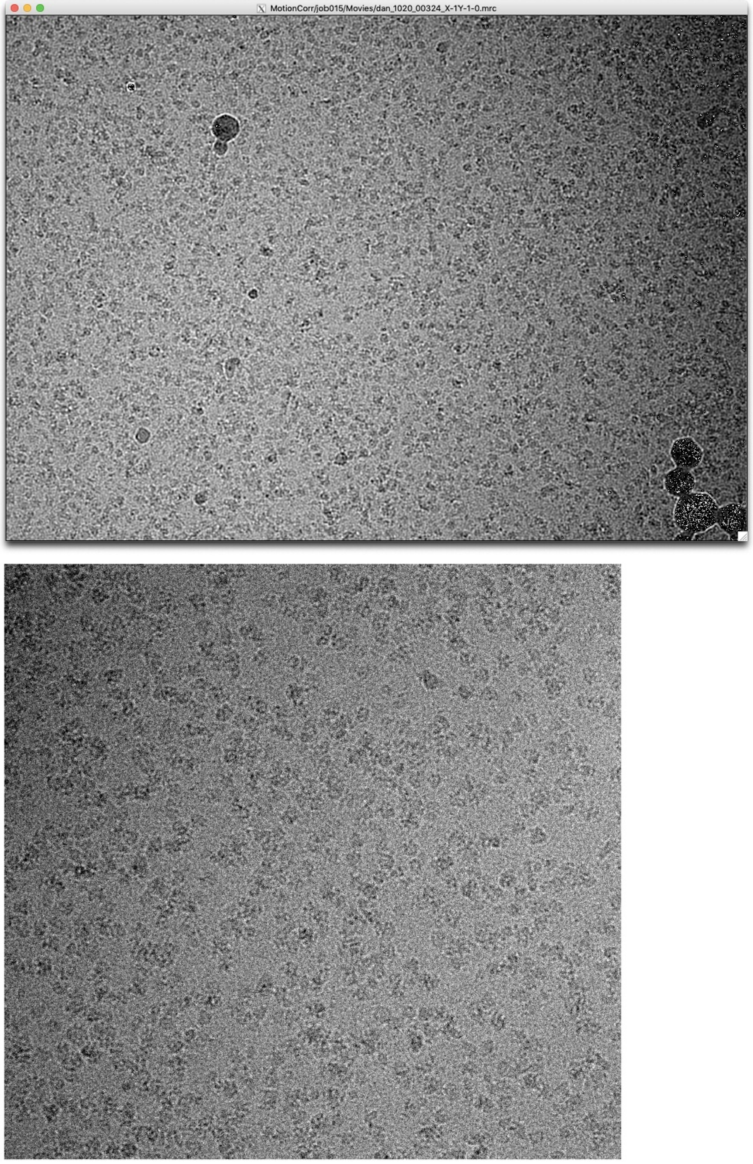

Supplementary Fig. 12. Representative cryo-EM images.

(a) Motion-corrected cryo-EM image for the OX_2R-mG_{sqiN}-scFv16 complex recorded on a Titan Krios microscope (FEI) operated at 300kV with a K3 direct electron detector (Gatan) at a nominal magnification of ×81,000.

(b) Motion-corrected cryo-EM image for the OX_2R-G_{i1}-scFv16 complex recorded on a Titan Krios microscope (FEI) operated at 300kV with a K2 direct electron detector (Gatan) at a nominal magnification of ×130,000.

a

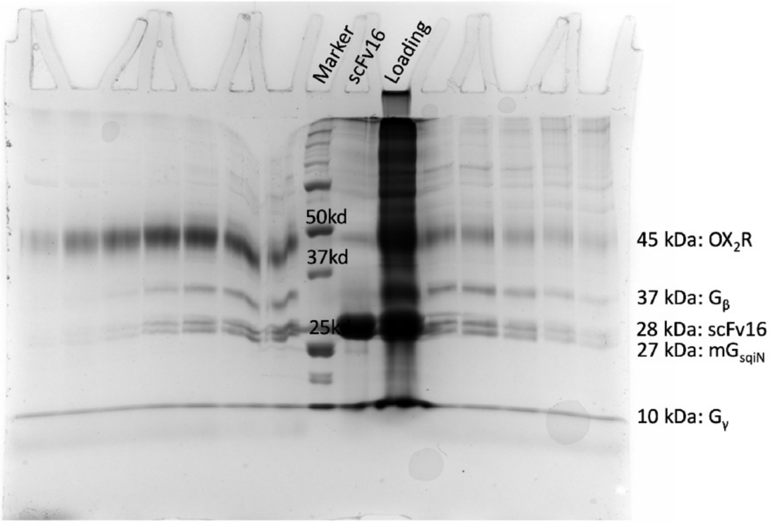

b

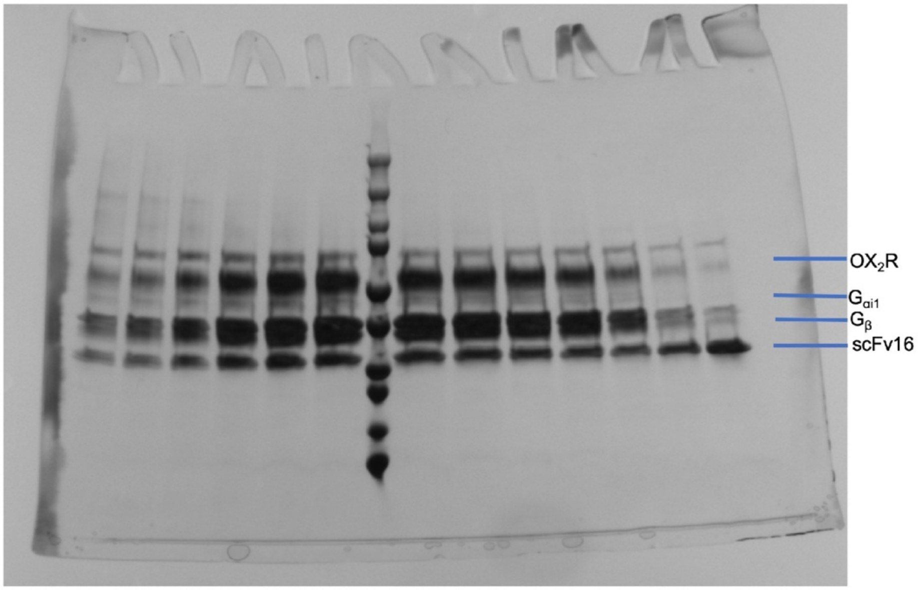

Supplementary Fig. 13. Uncropped gels.

(a) Uncropped gel of SEC (Size Exclusion Chromatography) fractions from the OX_2R-mG_{sqiN}-scFv16 purification. Cropped lane is shown in Supplementary Fig. 1b.

(b) Uncropped gel of SEC (Size Exclusion Chromatography) fractions from the OX_2R-G_{i1}-scFv16 purification. Cropped lane is shown in Supplementary Fig. 1d.

	OX_2R^{EM}-$mG_{\alpha sqiN}G_{\beta 1}G_{\lambda 2}$-scFv16 (EMDB-25399) (PDB 7SR8)	$OX2R^{EM}$-$DNG_{\alpha i1}G_{\beta 1}G_{\lambda 2}$-scFv16 (EMDB-25389) (PDB 7SQO)
**Data collection and processing**		
Magnification	81,000	130,000
Voltage (kV)	300	300
Electron exposure ($e^-/Å^2$)	64	50
Defocus range (μm)	1.6-2.6	1.3-1.9
Pixel size (Å)	1.08	1.04
Symmetry imposed	C1	C1
Initial particle images (no.)	8,929,023	2,735,296
Final particle images (no.)	155,362	332,173
Map resolution (Å)	3.27	3.12
FSC threshold	0.143	0.143
Map resolution range (Å)	3.1-3.8	3.1-3.8
**Refinement**		
Initial model used (PDB code)	6DDF, 6VMS, 6WHA	5WS3, 6OMM
Model Resolution (Å)	3.67	3.17
FSC threshold	0.5	0.5
Map sharpening *B* factor ($Å^2$)	-155	
Model composition		
Non-hydrogen atoms	9083	8626
Protein residues	1141 (9008 atoms)	1111 (8621 atoms)
Ligands	TAK-925 (1) OLA (4)	TAK-925 (1) OLA (4)
B factors ($Å^2$)		
Protein	70.7	107.7
Ligand	81.2	130.1
R.m.s deviations		
Bond lengths (Å)	0.003	0.003
Bond angles (°)	0.517	0.515
Validation		
MolProbity score	1.7	1.81
Clashscore	9.14	9.85
Poor rotamers (%)	0	0
Ramachandran plot		
Favored (%)	96.62	95.78
Allowed (%)	3.38	4.22
Disallowed (%)	0	0

Supplementary Table 1. Cryo-EM data collection and refinement statistics

Construct	Ligand	pEC_{50}	EC_{50} (nM)	E_{max}	n
OX_2R WT	TAK-925	8.1 ± 0.047	7.5	100	9
OX_2R T111A	TAK-925	5.7 ± 0.096****	2100	$53^{\dagger}$	3
OX_2R Q134A	TAK-925	not saturating			3
OX_2R N324A	TAK-925	7.8 ± 0.11*	18	$55^{\dagger}$	3
OX_2R WT	orexin B	8.2 ± 0.061	6.2	100	14
OX_2R T111A	orexin B	not saturating			3
OX_2R Q134A	orexin B	not saturating			3
OX_2R N324A	orexin B	not saturating			3
OX_2R T111S	TAK-925	6.6 ± 0.095****	230	$76^{\dagger}$	3
OX_2R T135A	TAK-925	6.2 ± 0.069****	580	$90^{\dagger}$	3
OX_2R T111S/T135A	TAK-925	5.4 ± 0.086****	3600	$78^{\dagger}$	3
OX_1R WT	TAK-925	not saturating			3
OX_1R S103T	TAK-925	5.3 ± 0.048****	5300	$130^{\&}$	3
OX_1R A127T	TAK-925	5.3 ± 0.052****	4700	$100^{\&}$	3
OX_1R S103T/A127T	TAK-925	6.5 ± 0.044****	300	100	3
$OX_2R^{1\text{-}406}$	TAK-925	8.3 ± 0.078	5.4	$100^{\dagger}$	6
$OX_2R^{1\text{-}406}$	orexinB	8.6 ± 0.083***	2.6	$100^{\$}$	7

Supplementary Table 2. Pharmacological parameters for IP accumulation assays. The pEC_{50} ± SEMs are shown from n independent experiments performed in duplicate. *P = 0.023, ***P = 0.0009, ****P < 0.0001, indicates pEC_{50} of mutant significantly different from that of OX_2R WT/Ligand by one-way ANOVA followed by Dunnett's test using GraphPad Prism 9 software. OX_1R TAK-925 data compared to OX_2R WT/TAK-925. $^{\dagger}E_{max}$ relative to OX_2R WT/TAK-925, $^{\$}E_{max}$ relative to OX_2R WT/orexin B, $^{\&}E_{max}$ relative to OX_1R S103T/A127T/TAK-925.

OX_2R	pEC50	EC50 (nM)
WT	6.9 ± 0.12	120
V114A	5.7 ± 0.068****	2200
W120A	<4.52	>30000
Q134A	<4.52	>30000
Q187A	5.5 ± 0.022****	3200
F227A	<4.52	>30000
F228A	6.2 ± 0.081****	600
Y232A	5.1 ± 0.037****	8500
Y317A	<4.52	>30000
K327A	5.6 ± 0.076****	2300
R328A	5.5 ± 0.018****	3000
F346A	6.5 ± 0.023**	350
H350A	5.3 ± 0.051****	4800
V353A	5.3 ± 0.015****	5500
Y354A	5.6 ± 0.024****	2400

Supplementary Table 3. β-arrestin recruitment activity of TAK-925 on OX_2R mutants. Data are the pEC50 ± SEMs from two or three independent experiments performed in quadruplicate. ***P* = 0.0019, *****P* < 0.0001, indicates pEC50 of mutant significantly different from that of WT by one-way ANOVA followed by Dunnett's test using GraphPad Prism software.

OX_2R	pEC50	EC50 (nM)
WT	8.4 ± 0.0024	4.1
V114A	6.8 ± 0.071****	150
W120A	< 6	>1000
Q134A	6.6 ± 0.12****	280
Q187A	7.5 ± 0.061***	29
F227A	< 6	>1000
F228A	7.9 ± 0.054*	13
Y232A	7.1 ± 0.064****	79
Y317A	6.9 ± 0.14****	120
K327A	6.9 ± 0.12****	120
R328A	6.2 ± 0.33****	590
F346A	6.9 ± 0.11****	140
H350A	6.7 ± 0.21****	200
V353A	7.6 ± 0.087**	24
Y354A	7.2 ± 0.11****	58

Supplementary Table 4. β-arrestin recruitment activity of OxB on OX_2R mutants. Data are the pEC50 ± SEMs from two or three independent experiments performed in quadruplicate. *P = 0.039, **P = 0.0028, ***P = 0.00025, ****P < 0.0001, indicates pEC50 of mutant significantly different from that of WT by one-way ANOVA followed by Dunnett's test using GraphPad Prism software.
