# Supplementary material for: Molecular mechanism of the wake-promoting agent TAK-925
Source: Nat Commun. 2022 May 25;13:2902. doi: 10.1038/s41467-022-30601-3 (PMC9133036; doi:10.1038/s41467-022-30601-3)
Supplement: Supplementary file 2 — Reporting Summary [file 41467_2022_30601_MOESM2_ESM.pdf]

Corresponding author(s): Mack Flinspach
Jef K. De Brabander
Daniel M. Rosenbaum

Last updated by author(s): Apr 10, 2022

Reporting Summary

Nature Portfolio wishes to improve the reproducibility of the work that we publish. This form provides structure for consistency and transparency in reporting. For further information on Nature Portfolio policies, see our Editorial Policies and the Editorial Policy Checklist.

Statistics

For all statistical analyses, confirm that the following items are present in the figure legend, table legend, main text, or Methods section.

n/a	Confirmed	
☐	☒	The exact sample size (*n*) for each experimental group/condition, given as a discrete number and unit of measurement
☐	☒	A statement on whether measurements were taken from distinct samples or whether the same sample was measured repeatedly
☐	☒	The statistical test(s) used AND whether they are one- or two-sided<br>*Only common tests should be described solely by name; describe more complex techniques in the Methods section.*
☒	☐	A description of all covariates tested
☒	☐	A description of any assumptions or corrections, such as tests of normality and adjustment for multiple comparisons
☐	☒	A full description of the statistical parameters including central tendency (e.g. means) or other basic estimates (e.g. regression coefficient) AND variation (e.g. standard deviation) or associated estimates of uncertainty (e.g. confidence intervals)
☐	☒	For null hypothesis testing, the test statistic (e.g. *F*, *t*, *r*) with confidence intervals, effect sizes, degrees of freedom and *P* value noted<br>*Give P values as exact values whenever suitable.*
☒	☐	For Bayesian analysis, information on the choice of priors and Markov chain Monte Carlo settings
☒	☐	For hierarchical and complex designs, identification of the appropriate level for tests and full reporting of outcomes
☒	☐	Estimates of effect sizes (e.g. Cohen's *d*, Pearson's *r*), indicating how they were calculated

Our web collection on statistics for biologists contains articles on many of the points above.

Software and code

Policy information about availability of computer code

Data collection	Cryo-EM data were collected using SerialEM v4.0 or Leginon v3.5.
Data analysis	Cryo-EM movie stacks were processed for beam-induced motion correction using MotionCor v1.4, and CTF correction was performed with GCTF v1.06. Cryo-EM data were analyzed and processed for 3D reconstruction using Relion v3.0 or cryoSPARC v2. Structures were built using Buccaneer v1.5 and Coot v0.9.2, and were refined using Phenix v1.19.2. Structural models were analyzed using the Molprobity webserver (molprobity.biochem.duke.edu). Structures were visualized and displayed using PyMOL v2.4.1 or UCSF Chimera v1.13.1. Quantum chemistry calculations were performed using NWChem v6.8.1. GPCR activation data were analyzed using GraphPad Prism v9.

For manuscripts utilizing custom algorithms or software that are central to the research but not yet described in published literature, software must be made available to editors and reviewers. We strongly encourage code deposition in a community repository (e.g. GitHub). See the Nature Portfolio guidelines for submitting code & software for further information.

Data

Policy information about availability of data

All manuscripts must include a data availability statement. This statement should provide the following information, where applicable:

- Accession codes, unique identifiers, or web links for publicly available datasets
- A description of any restrictions on data availability
- For clinical datasets or third party data, please ensure that the statement adheres to our policy

Structural data have been deposited in the Protein Data Bank (PDB) with coordinate accession numbers 7SQO and 7SR8 and maps have been deposited in the Electron Microscopy Data Bank (EMDB) with accession numbers EMD-25389 and EMD-25399. PDB entries 6DDF, 6WHA, 6VMS, 5WQC, and 6DDE were used in the

process of structural model building. PDB entries 7L1V, 4S0V, and 4ZJ8 were used for structural comparison and in figures. The pharmacological data generated in this study are compiled in the Source Data file provided with this paper.

Field-specific reporting

Please select the one below that is the best fit for your research. If you are not sure, read the appropriate sections before making your selection.

☒ Life sciences ☐ Behavioural & social sciences ☐ Ecological, evolutionary & environmental sciences

For a reference copy of the document with all sections, see nature.com/documents/nr-reporting-summary-flat.pdf

Life sciences study design

All studies must disclose on these points even when the disclosure is negative.

Sample size	For GPCR activation, studies were performed as three or more independent experiments, each done with multiple replicates. The size was chosen as sufficient to provide statistical significance in P-values according to one-way ANOVA, as described in Supplementary Tables 2-4. Sample size for cryo-EM imaging experiments (particle number) was chosen as sufficient to provide 3D reconstructions with map resolution according to gold-standard Fourier Shell Correlation 0.143.
Data exclusions	No data was excluded from the analyses.
Replication	All of the experiments in this study (GPCR activation, cryo-EM imaging) were replicated multiple times. GPCR activation studies were performed as 3 or more independent experiments, and cryo-EM analysis was performed on >2 million particles derived from >7000 high-resolution movie stack images. All other experiments (e.g. protein purifications) were performed independently three or more times, and all attempts at replication were successful.
Randomization	Cryo-EM data were collected and analyzed automatically without intervention, and processed using reference-free classification. No animal or clinical data were included in this study.
Blinding	Blinding was not applicable to structure determination or functional assays, since imaging data and functional data were collected and quantified automatically and without intervention.

Reporting for specific materials, systems and methods

We require information from authors about some types of materials, experimental systems and methods used in many studies. Here, indicate whether each material, system or method listed is relevant to your study. If you are not sure if a list item applies to your research, read the appropriate section before selecting a response.

Materials & experimental systems

n/a	Involved in the study	
☐	☒	Antibodies
☐	☒	Eukaryotic cell lines
☒	☐	Palaeontology and archaeology
☒	☐	Animals and other organisms
☒	☐	Human research participants
☒	☐	Clinical data
☒	☐	Dual use research of concern

Methods

n/a	Involved in the study	
☒	☐	ChIP-seq
☒	☐	Flow cytometry
☒	☐	MRI-based neuroimaging

Antibodies

Antibodies used	M1 ANTI-FLAG antibody (Sigma cat. F3040); HRP-coupled secondary antibody (Jackson ImmunoResearch cat. 715-035-150)
Validation	M1 ANTI-FLAG antibody: Multiple references and specifications detailed at https://www.sigmaaldrich.com/US/en/product/sigma/f3040.<br>HRP-coupled secondary antibody: Multiple references and specifications detailed at https://www.jacksonimmuno.com/catalog/products/715-035-150

Eukaryotic cell lines

Policy information about cell lines

Cell line source(s)	Sf9 Insect Cells (Expression Systems cat. 94-001S); HEK-293 cells (ATCC cat. CRL-1573)
Authentication	None of the cell lines were authenticated. Each was purchased from a commercial source - Sf9 Insect cells: references and

Authentication	specifications at https://expressionsystems.com/product/insect-cells; HEK-293 cells: references and specifications at https://www.atcc.org/products/crl-1573.
Mycoplasma contamination	The cell lines obtained commercially (as detailed above) were not tested for mycoplasma contamination.
Commonly misidentified lines (See ICLAC register)	NA
